# Supplementary material for: Photogrammetric reconstruction of 3D carpological collection in high resolution for plants authentication and species discovery
Source: PLoS One. 2022 Aug 4;17(8):e0270199. doi: 10.1371/journal.pone.0270199 (PMC9352034; doi:10.1371/journal.pone.0270199)
Supplement: S1 Table — The carpological specimens no., species name, carpological parts, size and voucher specimens no. (if any) of the carpological material were documented. (DOCX) [file pone.0270199.s001.docx]

**Table 4. 100 carpological materials adopted in this study for 3D model reconstruction.**

| **Carpological Specimen No.** | **Species Name** | **Carpological parts** | **Size** | **Voucher Specimen No.** |
| --- | --- | --- | --- | --- |
| PH001 | *Choerospondias axillaris* (Roxburgh) B. L. Burtt & A. W. Hill | Seed | 2.3 cm | NIL |
| PH002 | *Melastoma* sp. | Fruit | 2.6 cm | NIL |
| PH003 | *Xanthium* sp. | Fruit | 1.9 cm | NIL |
| PH004 | *Samanea saman* (Jacquin) Merrill | Fruit | 8 cm | NIL |
| PH005 | *Aleurites moluccana* (Linnaeus) Willdenow | Fruit | 4.7 cm | NIL |
| PH006 | *Alyxia sinensis* Champion ex Bentham | Seed | 1.1 cm | NIL |
| PH007 | *Datura* sp. | Fruit | 3.7 cm | NIL |
| PH008 | *Datura* sp. | Fruit & Seed | 3.2 cm | NIL |
| PH009 | *Engelhardia roxburghiana* Wallich | Fruit | 4.4cm | NIL |
| PH010 | *Trichosanthes* sp. | Seed | 0.8 cm | NIL |
| PH011 | Taxodiaceae plant | Strobilus | 3.8 cm | NIL |
| PH012 | *Acer* sp. | Fruit | 3.1 cm | NIL |
| PH013 | *Ficus pumila* Linnaeus | Fruit | 5.2 cm | NIL |
| PH014 | *Sapindus saponaria* Linnaeus | Fruit | 3.5 cm | NIL |
| PH015 | *Araucaria* sp. | Strobilus | 7.5 cm | NIL |
| PH016 | *Sterculia lanceolata* Cavanilles | Fruit | 11.5 cm | NIL |
| PH017 | *Heritiera littoralis* Aiton | Fruit | 5.7 cm | NIL |
| PH018 | *Heritiera littoralis* Aiton | Fruit | 5 cm | NIL |
| PH019 | *Castanopsis lamontii* Hance | Fruit | 4 cm | NIL |
| PH020 | *Luffa* sp. | Seed | 1.3 cm | NIL |
| PH021 | *Embelia* sp. | Seed | 0.5 cm | NIL |
| PH022 | *Casuarina equisetifolia* Linnaeus | Fruit | 2 cm | NIL |
| PH023 | *Polyspora axillaris* (Roxburgh ex Ker Gawler) Sweet | Fruit | 3.1 cm | NIL |
| PH024 | *Lithocarpus corneus* (Loureiro) Rehder | Fruit | 2.8 cm | NIL |
| PH025 | *Aegiceras corniculatum* (Linnaeus) Blanco | Fruit | 3.1 cm | NIL |
| PH026 | *Itea chinensis* Hooker & Arnott | Fruit | 0.8 cm | NIL |
| PH027 | *Lophostemon confertus* (R. Brown) Peter G. Wilson & J. T. Waterhouse | Fruit | 2 cm | NIL |
| PH028 | *Vicia faba* Linnaeus | Seed | 2.1 cm | NIL |
| PH029 | *Momordica cochinchinensis* (Loureiro) Sprengel | Seed | 2.4 cm | NIL |
| PH030 | *Thespesia populnea* (Linnaeus) Solander ex Corrêa | Seed | 1.1 cm | NIL |
| PH031 | *Trapa* sp. | Fruit | 6.3 cm | NIL |
| PH032 | *Dendrotrophe varians* (Blume) Miquel | Seed | 0.8 cm | NIL |
| PH033 | *Benincasa* sp. | Seed | 1.3 cm | NIL |
| PH034 | *Passiflora foetida* Linnaeus | Fruit | 4.3 cm | NIL |
| PH035 | *Calamus* sp*.* | Fruit | 2.1 cm | NIL |
| PH036 | *Canarium album* (Loureiro) Raeuschel | Fruit | 3 cm | NIL |
| PH037 | *Thevetia peruviana* (Persoon) K. Schumann | Fruit | 3.8 cm | NIL |
| PH038 | *Camptotheca acuminata* Decaisne | Fruit | 2.2 cm | NIL |
| PH039 | *Millettia* sp. | Fruit | 8.2 cm | NIL |
| PH040 | *Ochrosia elliptica* Labillardière | Seed | 4.1 cm | NIL |
| PH041 | *Laurocerasus phaeosticta* (Hance) C. K. Schneider | Seed | 0.8 cm | NIL |
| PH042 | *Pongamia pinnata* (Linnaeus) Merrill | Fruit | 5 cm | NIL |
| PH043 | *Pongamia pinnata* (Linnaeus) Merrill | Seed | 2 cm | NIL |
| PH044 | *Samanea saman* (Jacquin) Merrill | Fruit | 12.5 cm | NIL |
| PH045 | *Castanopsis lamontii* Hance | Fruit | 12 cm | NIL |
| PH046 | *Caesalpinia crista* Linnaeus | Fruit | 4.2 cm | NIL |
| PH047 | *Abutilon indicum* (Linnaeus) Sweet | Fruit | 3 cm | NIL |
| PH048 | *Helicteres angustifolia* Linnaeus | Fruit | 2 cm | NIL |
| PH049 | *Glochidion* sp. | Fruit | 1.1 cm | NIL |
| PH050 | *Alpinia* sp. | Fruit | 3.1 cm | NIL |
| PH051 | *Sterculia lanceolata* Cavanilles | Fruit | 8.6 cm | NIL |
| PH052 | *Sterculia lanceolata* Cavanilles | Fruit | 12.5 cm | NIL |
| PH053 | *Sterculia lanceolata* Cavanilles | Fruit | 12.6 cm | NIL |
| PH054 | *Lophostemon confertus* (R. Brown) Peter G. Wilson & J. T. Waterhouse | Fruit | 2.5 cm | NIL |
| PH055 | *Lophostemon confertus* (R. Brown) Peter G. Wilson & J. T. Waterhouse | Fruit | 2.6 cm | NIL |
| PH056 | *Pavetta hongkongensis* Bremekamp | Fruit | 1.1 cm | H. Y. Wong 008 |
| PH057 | *Pterospermum heterophyllum* Hance | Fruit | 6 cm | K. H. Wong 004 |
| PH058 | *Eucalyptus exserta* F. Mueller | Fruit | 2 cm | T. Y. Siu 571 |
| PH059 | *Avicennia marina* (Forsskål) Vierhapper | Fruit | 3.1 cm | D. T. W. Lau 252 |
| PH060 | *Mallotus peltatus* (Geiseler) Müller Argoviensis | Fruit | 1.3 cm | H. L. Wang 005 |
| PH061 | *Trema tomentosa* (Roxburgh) H. Hara | Fruit | 0.5 cm | H. L. Wang 006 |
| PH062 | *Memecylon ligustrifolium* Champion ex Bentham | Fruit | 1.6 cm | H. L. Wang 007 |
| PH063 | *Polyspora axillaris* (Roxburgh ex Ker Gawler) Sweet | Fruit | 3.4 cm | H. L. Wang 008 |
| PH064 | *Phyllanthus cochinchinensis* (Loureiro) Sprengel | Fruit | 0.8 cm | H. L. Wang 009 |
| PH065 | *Melastoma sanguineum* Sims | Fruit | 2.4 cm | H. L. Wang 010 |
| PH066 | *Helicteres angustifolia* Linnaeus | Fruit | 1.9 cm | H. L. Wang 011 |
| PH067 | *Styrax suberifolius* Hooker & Arnott | Fruit | 1.6 cm | H. L. Wang 012 |
| PH068 | *Camellia crapnelliana* Tutcher | Fruit | 9.8 cm | Rare and endangered plants (J. Y. Y Lau, D. T. W. Lau, K. W. Lam, S. W. Shek) 027 |
| PH069 | *Camellia oleifera* C. Abel | Fruit | 3.1 cm | Rare and endangered plants (D. T. W. Lau, J. Y. Y. Lau, S. W. Shek, K. W. Lam) 028 |
| PH070 | *Lithocarpus glaber* (Thunberg) Nakai | Fruit | 7 cm | T. Y. Siu 687 |
| PH071 | *Cyclobalanopsis pachyloma* (Seemen) Schottky | Fruit | 2.8 cm | T. Y. Siu 702 |
| PH072 | *Callerya nitida* (Bentham) R. Geesink Leiden | Fruit | 11.4 cm | H. L. Wang 014 |
| PH073 | *Wikstroemia indica* (Linnaeus) C. A. Meyer | Fruit | 1.1 cm | H. L. Wang 015 |
| PH074 | *Reevesia thyrsoidea* Lindley | Fruit | 3.4 cm | H. L. Wang 016 |
| PH075 | *Garcinia oblongifolia* Champion ex Bentham | Fruit | 3.7 cm | H. L. Wang 017 |
| PH076 | *Strychnos angustiflora* Bentham | Fruit | 2.6 cm | H. L. Wang 018 |
| PH077 | *Strychnos angustiflora* Bentham | Seed | 1.2 cm | H. L. Wang 018 |
| PH078 | *Dianella ensifolia* (Linnaeus) Redouté | Fruit | 1.1 cm | H. L. Wang 019 |
| PH079 | *Tetracera sarmentosa* (Linnaeus) Vahl | Fruit | 1.2 cm | H. L. Wang 020 |
| PH080 | *Uvaria calamistrata* Hance | Fruit | 6 cm | H. L. Wang 021 |
| PH081 | *Uvaria calamistrata* Hance | Seed | 0.9 cm | H. L. Wang 021 |
| PH082 | *Hibiscus tiliaceus* Linnaeus | Fruit | 3.3 cm | H. L. Wang 022 |
| PH083 | *Caesalpinia bonduc* (Linnaeus) Roxburgh | Fruit | 8.4 cm | H. L. Wang 023 |
| PH084 | *Caesalpinia bonduc* (Linnaeus) Roxburgh | Seed | 1.9 cm | H. L. Wang 023 |
| PH085 | *Vitex rotundifolia* Linnaeus f. | Fruit | 0.7 cm | H. L. Wang 024 |
| PH086 | *Canavalia* sp. | Fruit | 11 cm | H. L. Wang 025 |
| PH087 | *Canavalia* sp. | Seed | 1.8 cm | H. L. Wang 025 |
| PH088 | *Ricinus communis* Linnaeus | Fruit | 2 cm | H. L. Wang 026 |
| PH089 | *Ipomoea pes-caprae* (Linnaeus) R. Brown | Fruit | 2.7 cm | H. L. Wang 027 |
| PH090 | *Psychotria asiatica* Linnaeus | Fruit | 0.9 cm | H. L. Wang 028 |
| PH091 | *Clerodendrum fortunatum* Linnaeus | Fruit | 2.2 cm | H. L. Wang 029 |
| PH092 | *Brucea javanica* (Linnaeus) Merrill | Fruit | 1.7 cm | H. L. Wang 030 |
| PH093 | *Antidesma bunius* (Linnaeus) Sprengel | Fruit | 2.5 cm | H. L. Wang 031 |
| PH094 | *Gnetum luofuense* C. Y. Cheng | Seed | 5.6 cm | M. C. Li 071 |
| PH095 | *Antidesma japonicum* Siebold & Zuccarini | Fruit | 2.2 cm | M. C. Li 076 |
| PH096 | *Castanopsis eyrei* (Champion ex Bentham) Tutcher | Fruit | 4.1 cm | M. C. Li 077 |
| PH097 | *Aidia canthioides* (Champion ex Bentham) Masamune | Fruit | 1.5 cm | H. L. Wang 032 |
| PH098 | *Mallotus paniculatus* (Lamarck) Müller | Fruit | 1 cm | H. L. Wang 033 |
| PH099 | *Strychnos umbellata* (Loureiro) Merrill | Fruit | 1.1 cm | H. L. Wang 034 |
| PH100 | *Strychnos umbellata* (Loureiro) Merrill | Seed | 0.9 cm | H. L. Wang 034 |
