# Supplementary material for: Photogrammetric reconstruction of 3D carpological collection in high resolution for plants authentication and species discovery
Source: PLoS One. 2022 Aug 4;17(8):e0270199. doi: 10.1371/journal.pone.0270199 (PMC9352034; doi:10.1371/journal.pone.0270199)

PLOS ONE

Photogrammetric reconstruction of 3D carpological collection in high resolution for plants authentication and species discovery

--Manuscript Draft--

**Manuscript Number:**	PONE-D-22-00891
**Article Type:**	Research Article
**Full Title:**	Photogrammetric reconstruction of 3D carpological collection in high resolution for plants authentication and species discovery
**Short Title:**	Photogrammetric reconstruction of 3D carpological collection
**Corresponding Author:**	David Tai-Wai LAU, Ph.D.<br>The Chinese University of Hong Kong<br>HONG KONG
**Keywords:**	Photogrammetry; Herbarium; Carpological collection; 3D models; Plant specimens
**Abstract:**	This study provides an accurate and efficient method to reconstruct detailed and high-resolution digital 3D models of carpological materials by photogrammetric method, in which only about 100 to 150 images are required for each model reconstruction. The 3D models reflect the realistic morphology and genuine color of the carpological materials. The 3D models are scaled to represent the true size of the materials even as small as 3 mm in diameter. The interfaces are interactive, in which the 3D models can be rotated in 360° to observe the structures and be zoomed to inspect the macroscopic details. This new platform is a prerequisite of developing a virtual herbarium of carpological collection which is thus the most important to botanical authentication and education.
**Order of Authors:**	Ho Lam Wang
	Tin Hang Wong
	Yiu Man Chan
	Yat Sum Cheng
	David Tai-Wai LAU, Ph.D.
**Additional Information:**	
**Question**	**Response**
**Financial Disclosure**<br><br>Enter a financial disclosure statement that describes the sources of funding for the work included in this submission. Review the submission guidelines for detailed requirements. View published research articles from *PLOS ONE* for specific examples.<br><br>This statement is required for submission and **will appear in the published article** if the submission is accepted. Please make sure it is accurate.	This study was funded by Wu Jieh Yee Charitable Foundation Limited.<br><br>The funders had no role in study design, data collection and analysis, decision to publish, or preparation of the manuscript.

Powered by Editorial Manager® and ProduXion Manager® from Aries Systems Corporation

**Unfunded studies**<br>Enter: *The author(s) received no specific funding for this work.*<br><br>**Funded studies**<br>Enter a statement with the following details:<br>• Initials of the authors who received each award<br>• Grant numbers awarded to each author<br>• The full name of each funder<br>• URL of each funder website<br>• Did the sponsors or funders play any role in the study design, data collection and analysis, decision to publish, or preparation of the manuscript?<br>• **NO** - Include this sentence at the end of your statement: *The funders had no role in study design, data collection and analysis, decision to publish, or preparation of the manuscript.*<br>• **YES** - Specify the role(s) played.<br><br>* typeset	
**Competing Interests**<br><br>Use the instructions below to enter a competing interest statement for this submission. On behalf of all authors, disclose any competing interests that could be perceived to bias this work—acknowledging all financial support and any other relevant financial or non-financial competing interests.<br><br>This statement is required for submission and **will appear in the published article** if the submission is accepted. Please make sure it is accurate and that any funding sources listed in your Funding Information later in the submission form are also declared in your Financial Disclosure statement.<br><br>View published research articles from *PLOS ONE* for specific examples.	The authors have declared that no competing interests exist.

**NO authors have competing interests**<br><br>Enter: *The authors have declared that no competing interests exist.*<br><br>**Authors with competing interests**<br><br>Enter competing interest details beginning with this statement:<br><br>*I have read the journal's policy and the authors of this manuscript have the following competing interests: [insert competing interests here]*<br><br>* typeset	
**Ethics Statement**<br><br>Enter an ethics statement for this submission. This statement is required if the study involved:<br><br>• Human participants<br>• Human specimens or tissue<br>• Vertebrate animals or cephalopods<br>• Vertebrate embryos or tissues<br>• Field research<br><br>Write "N/A" if the submission does not require an ethics statement.<br><br>General guidance is provided below. Consult the submission guidelines for detailed instructions. **Make sure that all information entered here is included in the Methods section of the manuscript.**	N/A

**Format for specific study types**<br><br>**Human Subject Research (involving human participants and/or tissue)**<br>• Give the name of the institutional review board or ethics committee that approved the study<br>• Include the approval number and/or a statement indicating approval of this research<br>• Indicate the form of consent obtained (written/oral) or the reason that consent was not obtained (e.g. the data were analyzed anonymously)<br><br>**Animal Research (involving vertebrate animals, embryos or tissues)**<br>• Provide the name of the Institutional Animal Care and Use Committee (IACUC) or other relevant ethics board that reviewed the study protocol, and indicate whether they approved this research or granted a formal waiver of ethical approval<br>• Include an approval number if one was obtained<br>• If the study involved *non-human primates*, add *additional details* about animal welfare and steps taken to ameliorate suffering<br>• If anesthesia, euthanasia, or any kind of animal sacrifice is part of the study, include briefly which substances and/or methods were applied<br><br>**Field Research**<br><br>Include the following details if this study involves the collection of plant, animal, or other materials from a natural setting:<br>• Field permit number<br>• Name of the institution or relevant body that granted permission	
**Data Availability**<br><br>Authors are required to make all data underlying the findings described fully available, without restriction, and from the time of publication. PLOS allows rare exceptions to address legal and ethical concerns. See the PLOS Data Policy and FAQ for detailed information.	Yes - all data are fully available without restriction

A Data Availability Statement describing where the data can be found is required at submission. Your answers to this question constitute the Data Availability Statement and **will be published in the article**, if accepted.<br><br>**Important:** Stating 'data available on request from the author' is not sufficient. If your data are only available upon request, select 'No' for the first question and explain your exceptional situation in the text box.<br><br>Do the authors confirm that all data underlying the findings described in their manuscript are fully available without restriction?	
**Describe where the data may be found in full sentences. If you are copying our sample text, replace any instances of XXX with the appropriate details.**<br><br>• If the data are **held or will be held in a public repository**, include URLs, accession numbers or DOIs. If this information will only be available after acceptance, indicate this by ticking the box below. For example: *All XXX files are available from the XXX database (accession number(s) XXX, XXX.).*<br>• If the data are all contained **within the manuscript and/or Supporting Information files**, enter the following: *All relevant data are within the manuscript and its Supporting Information files.*<br>• If neither of these applies but you are able to provide **details of access elsewhere**, with or without limitations, please do so. For example:<br><br>*Data cannot be shared publicly because of [XXX]. Data are available from the XXX Institutional Data Access / Ethics Committee (contact via XXX) for researchers who meet the criteria for access to confidential data.*<br><br>*The data underlying the results presented in the study are available from (include the name of the third party*	All relevant data are within the manuscript and its Supporting Information files.

and contact information or URL).<br>• This text is appropriate if the data are owned by a third party and authors do not have permission to share the data.<br><br>* typeset	
Additional data availability information:	

Click here to access/download;Manuscript;Main Text.docx

Photogrammetric reconstruction of 3D carpological collection in high resolution for plants authentication and species discovery

Short title: Photogrammetric reconstruction of 3D carpological collection

Ho Lam Wang[1], Tin Hang Wong[1], Yiu Man Chan[1], Yat Sum Cheng[1], Tai Wai David Lau[1*]

[1] Shiu-Ying Hu Herbarium, School of Life Sciences, The Chinese University of Hong Kong, Hong Kong Special Administrative Region, the People's Republic of China

* Corresponding author
E-mail: lautaiwai@cuhk.edu.hk (DTWL)

Abstract

This study provides an accurate and efficient method to reconstruct detailed and high-resolution digital 3D models of carpological materials by photogrammetric method, in which only about 100 to 150 images are required for each model reconstruction. The 3D models reflect the realistic morphology and genuine color of the carpological materials. The 3D models are scaled to represent the true size of the materials even as small as 3 mm in diameter. The interfaces are interactive, in which the 3D models can be rotated in 360° to observe the structures and be zoomed to inspect the macroscopic details. This new platform is a prerequisite of developing a virtual herbarium of carpological collection which is thus the most important to botanical authentication and education.

Introduction

Mass digitization has become a trend in the past years to document natural history collections and herbarium archives which facilitated the knowledge transfer of biodiversity information [1-3]. 2D scanning is one of the major digitization methods in many herbaria and museums, in which specimens are captured as high-quality images [4-7]. However, 2D images are usually plain and fixed which cannot show a specimen from all angles of view. Also, some structures cannot be observed from the images due to overlapping [8].

Technological advancement has attracted much interest in the development of 3D digitization of natural history collection. The technique assists the transformation of natural history collection into 3D models which provides more information than those from the 2D counterpart. Laser scanning is one of the common methods to create 3D models of specimens with detailed external structures [9-11]. Computed tomography (CT) scanning is another method by using X-ray to penetrate specimens and document the internal structures in a pile of images, which can be stacked into 3D models [12,13]. However, the genuine color of the specimens cannot be reflected on the 3D models reconstructed by laser or CT scanning, so this important phenotypic character is missing.

Photogrammetry has been applied in the 3D digitization of biological specimens by capturing images of the specimens from different angles. This method was firstly adopted on studying insect specimens [14-16], and later be extended to mammal and bird specimens [17-19]. The 3D models not only reflect the external structures of the specimens, but also reveal their color, which could aid for authentication and conservation of the animal species. Photogrammetry was further utilized to record live animals by multiple cameras to instantaneously capture images of the animals and then succeeded to the 3D model reconstruction [20]. Such approaches demonstrated how to digitize live species and natural history collections into 3D models by photogrammetry, which provide references for species documentation.

In the meantime, photogrammetry has been applied in various botanical studies. 3D models of live agricultural plants were reconstructed with photogrammetric methods, so as to estimate their growth rate and biomass, from which were the parameters were found to be highly accurate and precise [21-25]. Furthermore, photogrammetry also provided a low-cost and non-destructive alternative to document live plant records [26,27]. Despite the wide application of digitization in animal specimens and agricultural plants, the photogrammetric reconstruction of plant specimens, especially

their carpological parts, is still very rudimentary.

This research study outlined a tailor-made photogrammetric platform and a standardized methodology for digitizing carpological materials into 3D models. The settings are relatively stable and replicable. 3D models of 100 carpological materials had been reconstructed in this study. Results consolidated that this platform and method are feasible and replicable in mass digitization of carpological materials. The 3D models had also been uploaded to a newly developed online database for researchers and the public. The limitations of this platform were also discussed with proposed solutions. In future, the new platform can be applied to create virtual carpological herbaria in different regions for the purposes of research and education.

Materials and methods

Trial usage of conventional 3D scanner

At first, an industrial 3D scanning platform was used to reconstruct 3D models of carpological materials. The platform consisted of a digital 3D scanner and a turntable which were synchronized by a computer software under a same brand. A circular paper with reference points was placed on the turntable. The carpological material could be placed in the middle of the turntable, in which the scanning process was done automatically by the software. Although the reconstruction process was fully automated, the 3D model did not fulfill our research aims as it could not reflect the genuine color, resolution and structures of the carpological material. Hence, we began to build up our tailor-made platform as below.

Selection of digital camera body

In order to optimize the image quality and facilitate the image capturing process, several types of cameras had been examined, including digital camera, digital single-lens reflex camera (DSLR) and mirrorless camera (Table 1). The digital camera Olympus TG-4 with a built-in microscope mode was firstly used in the experiment. Close-up images of insect specimens and small flowers were captured by using the TG-4, but the image quality could not be further analyzed because of their insufficient effective

pixels (16 megapixels) and image resolution (4608 x 3456 pixels). Since the image resolution would significantly affect the quality of the 3D models, two DSLR cameras, namely Canon EOS 80D and EOS 5D SR were tried to achieve high image resolution up to 6K and 8K respectively. Both DSLR cameras were equipped with a touchable live-view panel, so as to minimize shutter-induced vibration. The reconstructed 3D models showed the macroscopic details of the carpological material. However, both DSLR cameras could not sharply focus on objects smaller than 5 mm in diameter in live-view shooting mode. The mirrorless camera, Canon EOS R5 with 8K-resolution was finally adopted because it performed better than the other two DSLR cameras, whereas it was found to provide high quality images of very small objects down to 3 mm under an efficient auto-focusing response.

Table 1. Specification of the camera bodies used in the experimental trials.

	Olympus TG-4	Canon EOS 80D	Canon EOS 5D SR	Canon EOS R5
Camera type	Digital	DSLR	DSLR	Mirrorless
Effective pixels (megapixels)	16	24.2	50.6	45
Max. image resolution	4608 x 3456	6000 x 4000	8688 x 5792	8192 x 5464

(pixels)				
Touch screen	No	Yes	Yes	Yes

Selection of camera lenses

In the research of the magnification and image quality, three camera lenses had be[illegible] examined, including Canon MP-E 65mm f/2.8 1-5X Macro Photo, Canon EF 35mm f/2 IS USM and Canon EF 100mm f/2.8L Macro IS USM (Table 2). The MP-E 65mm was expected to be better because of its 5 times magnification, however, the MP-E 65mm could not be operated by auto-focusing system, so its manual operation would largely increase the image capturing time. Hence, it was not practical and efficient for mass production of 3D models. The EF 35mm is a wide-angle lens with auto-focus mode, which could be used to capture images of larger carpological materials in good quality. It was noted that the EF 35mm has only a maximum magnification of 0.24 times which could not show the macroscopic details. The EF 100mm is a 1:1 macro lens with auto-focus mode that was found to be satisfactory for documenting both tiny and large carpological materials. Furthermore, the images under high magnification could still show very clear macroscopic structures of the materials. Finally, the EF 100mm macro lens was selected as the operational lens.

Table 2. Specificatio[illegible]f the camera lenses used in the experimental trials.

	Canon EF 35mm f/2 IS USM	Canon MP-E 65mm f/2.8 1-5X Macro Photo	Canon EF 100mm f/2.8L Macro IS USM
Lens type	Wide angle lens	Macro lens	Macro lens
Max. magnification (x)	0.24	5	1
Focus mode	Auto/Manual	Manual	Auto/Manual

Adjustment of light source

Lighting system of different configurations were conducted to ensure the 3D models reflecting the genuine color of the carpological materials with minimized image noise. Firstly, two lamps of no specification in light intensity and color temperature were placed on top of the carpological material. It was noted that the bottom part of the 3D model was darker than the upper part. Image noise was also relatively high because of ISO 6400 adoption under low light intensity of the LED lamps. Moreover, the 3D models could not show the genuine color because of unspecific color temperature. Hence, three light-plates of Phottix M200R RGB Light were used instead. The light intensity and the color temperature were adjusted to 100% and 5600K respectively to mimic the daylight spectrum. The light-plates were placed at both sides and the top,

that facing directly towards the carpological material. The images captured and the 3D models reconstructed from this lighting settings could truly reflect the genuine color of the carpological material, and the image quality was very high because of minimized image noise.

Software selection for photogrammetry and 3D model reconstruction

There are many photogrammetric software products available in the market. A number of trials were conducted to select a suitable one for researching our 3D reconstruction of carpological materials. The first completed 3D model in this study was a fruit of *Aleurites moluccana* (Linnaeus) Willdenow (PH005), reconstructed by using the AliceVision Meshroom (version 2021.1.0; https://alicevision.org/#meshroom). A total of 128 images were captured from four vertical angles towards the object (20°, 0°, -20° and -25°) with a horizontal rotation interval of 11.25° until the turntable completely rotated for 360°. 8K-resolution images were firstly used for reconstruction, but the 3D model was incomplete. It was likely because the 8K-resolution images are too i[illegible]rmative that is out of the software analytical range. Hence, the images were trimmed down to 4320 x 2880 pixels for matching the software capacity in order to reconstruct the 3D model. The whole experimental time was about three hours from image capturing to model

reconstruction. The 3D model could show the shape and structure of the carpological material, but the model color was dimmer and the texture was blurred when comparing to the sample material. Additional trials of 3D model reconstruction were carried out by the AliceVision Meshroom, but the software processing time was too long that might not be favorable to mass production. On the other hand, image trimmed to lower resolution would lose some characteristic details that is not scientific for species authentication.

Afterwards, “3D Zephyr Free” (version 6.006; https://www.3dflow.net/3df-zephyr-free/), a trial version of the 3D Zephyr series was researched in the 3D model reconstruction, but it has a limitation of 50 images on each reconstruction analysis. Therefore, only 48 out of 128 images were selected, consisting three sets of 16 images from three vertical angles (20°, 0° and -20°) with horizontal rotation interval of 22.5°. Unexpectedly, the reconstruction process finished within 30 minutes, and the quality of the 3D model was better than all previous models. They kept the genuine color and the texture, and showed the macroscopic details. Since the fruit of *A. moluccana* (PH005) had a relatively simple structure, further trials were conducted using a fruit of *Xanthium* sp. (PH003) with numerous of spines, to test the software capacity on analyzing more complex structures. 48 images of PH003 were captured using the same

configuration, but the reconstruction was not successful. It was possibly because PH003 was highly symmetric, and 48 images from three angles were not distinctive enough to be recognized by the software.

It was also noted that the number of images would directly determine the quality and the completeness of the 3D models, as well as the computing time of analysis. Importing more images to the software might provide more information for the reconstruction, which enhances the quality and the completeness of the 3D models, but the computing time would be much longer. For example, 3 days were required to analyze 700 images in some of our early trials. In contrast, importing less images to the software could reduce the computing time, but the 3D models might be incomplete or at low quality. Therefore, we have been experimenting on various image number in the new software to create the best quality of the 3D models with good efficiency.

The “3D Zephyr Lite” (version 6.006-6.010; https://www.3dflow.net/3df-zephyr-photogrammetry-software/), an advanced version of the software with a maximum input of 500 images on each reconstruction was purchased in this regard. Basically, a total of 96 images of PH003 were captured from three vertical angles (20°, 0° and -20°)

with a horizontal rotation interval of 11.25° were imported to the software. For more complicated materials, more images from wider vertical angles (e.g. 35° and -35°) were required to produce a complete and high-quality 3D model. The procedures were repeatedly validated by applying in more than 50 carpological materials of different shapes and structures. The results further confirmed the usage of “3D Zephyr Lite” as software for photogrammetric reconstruction in our platform.

Results

New integration and establishment of standardized photogrammetric platform

After experimentation and optimization of the equipment settings and their usage, an image capturing platform and a standardized pipeline for 3D model reconstruction were established, which enable an efficient 3D digitization of carpological materials. The details are described as follows.

Apparatus and configuration

The settings of the image capturing station are shown in Fig 1. A mirrorless camera Canon EOS R5 with a macro lens Canon EF100mm f/2.8L Macro IS USM using a mount adapter EF-EOS R was used to capture images of carpological materials. The camera was fixed on a tripod, consisting of a Manfrotto 190D stand, a Manfrotto XPRO geared three-way pan/tilt tripod head, and a Fotomate LP-01 macro-turning long-type rail slider. A Phottix tabletop portable photo studio was used to provide a matte background of either white or black to distinguish the carpological materials of different colors.

Fig 1. Set up of the photogrammetric station. A mirrorless camera Canon EOS R5 with

a macro lens (Canon EF 100mm f/2.8L Macro IS USM) is mounted on a tripod. The carpological material is affixed on a pin which is placed in the middle of a turntable. Three light-plates are placed on the top, left and right side of the carpological material. A portable photo studio is used as background.

The carpological material was attached firmly on a pin with diameter corresponding to the material size. The pin was fixed in vertical orientation on a plastic container. A tailor-made triangular marker was affixed through the pin as a size reference. The lateral faces of the marker had scale rulers and specific herbarium logo which aid for accurate image recognition by the software. The carpological material with the marker were then placed at the middle of a ComXim electric turntable MT200RL20, which enabled image capturing from 360° view. Three Phottix M200R RGB Light were installed on top, left and right sides of the carpological material from a distance of 15 to 20 cm, so as to provide stable and well-dispersed light source.

Image capturing

The settings of the camera were kept consistent throughout the image capturing procedures. The adopted configuration was shown in Table 3. The aperture was fixed at f/13. The light sensitivity (ISO) of the camera was adjusted according to the

morphological structures and the color of the carpological material. The settings of the light-plates were also remained consistent (CCT mode; color temperature 5600 K). The camera and the macro lens were adjusted to auto-focus mode and the images were captured in live-view shooting mode.

Table 3. Configuration of the camera Canon EOS R5 for image capturing.

Camera mode	Manual
Shutter speed (s)	1/100
Aperture	f13
Light sensitivity	ISO 800 - 2000
Color temperature (K)	5600
Image resolution (pixels)	8192 x 5464

The images were captured basically from three vertical angles to the carpological materials (any angle between 20° to 25°, 0° and any angle between -20° to -25°), with a horizontal rotation of 11.25° until a cycle was completed. Therefore, a total of 96 images were captured for each reconstruction (Fig 2). For carpological materials of very complicated structures, additional images from vertical angles 35° and -35° were captured to acquire more information of the carpological material. At each angle, the camera was adjusted to the minimum focusing distance from the carpological material,

or until the carpological material and the marker appeared in full size on the camera monitor by moving the rail slider. It is important to capture the carpological material together with the marker in order to provide a real-time reference of the material size.

Fig 2. Camera position estimated by 3D Zephyr Lite. 96 images of the fruit of *Ficus pumila* Linnaeus (PH013) were captured from 20°, 0° and -20° vertically towards the carpological material, with at a horizontal rotation interval of 11.25°. The red triangles represent the camera positions estimated by 3D Zephyr Lite.

Image analysis and 3D model reconstruction

The images were analyzed by a software 3DF Zephyr Lite under a high-ranked computing system (CPU: Alienware Aurora R11 with an 11th Gen Intel® Core ™ i7 11700KF; GPU: NVIDIA GeForce RTX 3090). For the software settings, the category and presets for camera orientation were “General” and “Deep” respectively. “General” and “High details” were selected for dense point cloud creation, surface reconstruction, and texturing. The software would show the number of selected images for the model reconstruction after internal calculation, and the data point cloud of the 3D model and the positions of image capturing would be previewed.

The reconstructed 3D model consisted of two major components, namely “Mesh” and “Texture” (Fig 3). Mesh referred to a monochrome solid model composed of many polygons, while texture refers to the surface color of the model. The texture was exported as a separated JPG/PNG file after the reconstruction, which was automatically applied to the structure of the mesh file. Hence, the 3D model was composed of a mesh covered by a layer of texture.

Fig 3. Composition of a 3D model reconstructed by photogrammetric platform. (a) A carpological material of the fruit of *Aleurites moluccana* (Linnaeus) Willdenow (PH005). (b) Texture of the 3D model extracted by 3DF Zephyr Lite. (c) A 3D model with texture to show the genuine color of PH005. (d) A 3D model without texture to show the plain mesh of PH005.

3-D model editing

During model reconstruction, some unwanted meshes such as the background and the pin would also be reconstructed. Therefore, the 3D model was exported as textured mesh and imported to another open software Blender (version 2.93; https://www.blender.org/) for manual editing. The unwanted structures could be deleted while the specimen with the scale bar were kept in the 3D model. The edited

3D model was saved as a new file of the final product.

Establishment of "Virtual Carpological Herbarium of Fruits and Seeds" in the Shiu-Ying Hu Herbarium

In this study, 3D models of 100 carpological materials were reconstructed (Table 4). Each 3D model was assigned with a code (e.g. PH001) for archival record in herbarium. The 3D models PH001 to PH055 were derived from carpological materials during the method development stage. The successful reconstruction of these 55 models had further consolidated a complete workflow and pipeline of the photogrammetric platform (Fig 4). The 3D models from PH056 onwards were fresh carpological parts of newly collected voucher specimens. All the 3D models were uploaded to an open online database (https://syhuherbarium.sls.cuhk.edu.hk/collections/3d-specimen/; Username: syhuherbarium; Password: @CUHK).

Table 4. 100 carpological materials adopt[illegible]n this study for 3D model reconstruction.

Carpological Specimen No.	Species Name	Carpological parts	Size	Voucher Specimen No.
PH001	*Choerospondias axillaris* (Roxburgh) B. L. Burtt & A.	Seed	2.3 cm	NIL

	W. Hill			
PH002	*Melastoma* sp.	Fruit	2.6 cm	NIL
PH003	*Xanthium* sp.	Fruit	1.9 cm	NIL
PH004	*Samanea saman* (Jacquin) Merrill	Fruit	8 cm	NIL
PH005	*Aleurites moluccana* (Linnaeus) Willdenow	Fruit	4.7 cm	NIL
PH006	*Alyxia sinensis* Champion ex Bentham	Seed	1.1 cm	NIL
PH007	*Datura* sp.	Fruit	3.7 cm	NIL
PH008	*Datura* sp.	Fruit & Seed	3.2 cm	NIL
PH009	*Engelhardia roxburghiana* Wallich	Fruit	4.4cm	NIL
PH010	*Trichosanthes* sp.	Seed	0.8 cm	NIL
PH011	Taxodiaceae plant	Strobilus	3.8 cm	NIL
PH012	*Acer* sp.	Fruit	3.1 cm	NIL
PH013	*Ficus pumila* Linnaeus	Fruit	5.2 cm	NIL
PH014	*Sapindus saponaria* Linnaeus	Fruit	3.5 cm	NIL

PH015	*Araucaria* sp.	Strobilus	7.5 cm	NIL
PH016	*Sterculia lanceolata* Cavanilles	Fruit	11.5 cm	NIL
PH017	*Heritiera littoralis* Aiton	Fruit	5.7 cm	NIL
PH018	*Heritiera littoralis* Aiton	Fruit	5 cm	NIL
PH019	*Castanopsis lamontii* Hance	Fruit	4 cm	NIL
PH020	*Luffa* sp.	Seed	1.3 cm	NIL
PH021	*Embelia* sp.	Seed	0.5 cm	NIL
PH022	*Casuarina equisetifolia* Linnaeus	Fruit	2 cm	NIL
PH023	*Polyspora axillaris* (Roxburgh ex Ker Gawler) Sweet	Fruit	3.1 cm	NIL
PH024	*Lithocarpus corneus* (Loureiro) Rehder	Fruit	2.8 cm	NIL
PH025	*Aegiceras corniculatum* (Linnaeus) Blanco	Fruit	3.1 cm	NIL
PH026	*Itea chinensis* Hooker & Arnott	Fruit	0.8 cm	NIL

PH027	*Lophostemon confertus* (R. Brown) Peter G. Wilson & J. T. Waterhouse	Fruit	2 cm	NIL
PH028	*Vicia faba* Linnaeus	Seed	2.1 cm	NIL
PH029	*Momordica cochinchinensis* (Loureiro) Sprengel	Seed	2.4 cm	NIL
PH030	*Thespesia populnea* (Linnaeus) Solander ex Corrêa	Seed	1.1 cm	NIL
PH031	*Trapa* sp.	Fruit	6.3 cm	NIL
PH032	*Dendrotrophe varians* (Blume) Miquel	Seed	0.8 cm	NIL
PH033	*Benincasa* sp.	Seed	1.3 cm	NIL
PH034	*Passiflora foetida* Linnaeus	Fruit	4.3 cm	NIL
PH035	*Calamus* sp.	Fruit	2.1 cm	NIL
PH036	*Canarium album* (Loureiro) Raeuschel	Fruit	3 cm	NIL
PH037	*Thevetia peruviana* (Persoon) K. Schumann	Fruit	3.8 cm	NIL

PH038	*Camptotheca acuminata* Decaisne	Fruit	2.2 cm	NIL
PH039	*Millettia* sp.	Fruit	8.2 cm	NIL
PH040	*Ochrosia elliptica* Labillardière	Seed	4.1 cm	NIL
PH041	*Laurocerasus phaeosticta* (Hance) C. K. Schneider	Seed	0.8 cm	NIL
PH042	*Pongamia pinnata* (Linnaeus) Merrill	Fruit	5 cm	NIL
PH043	*Pongamia pinnata* (Linnaeus) Merrill	Seed	2 cm	NIL
PH044	*Samanea saman* (Jacquin) Merrill	Fruit	12.5 cm	NIL
PH045	*Castanopsis lamontii* Hance	Fruit	12 cm	NIL
PH046	*Caesalpinia crista* Linnaeus	Fruit	4.2 cm	NIL
PH047	*Abutilon indicum* (Linnaeus) Sweet	Fruit	3 cm	NIL
PH048	*Helicteres angustifolia* Linnaeus	Fruit	2 cm	NIL

PH049	*Glochidion* sp.	Fruit	1.1 cm	NIL
PH050	*Alpinia* sp.	Fruit	3.1 cm	NIL
PH051	*Sterculia lanceolata* Cavanilles	Fruit	8.6 cm	NIL
PH052	*Sterculia lanceolata* Cavanilles	Fruit	12.5 cm	NIL
PH053	*Sterculia lanceolata* Cavanilles	Fruit	12.6 cm	NIL
PH054	*Lophostemon confertus* (R. Brown) Peter G. Wilson & J. T. Waterhouse	Fruit	2.5 cm	NIL
PH055	*Lophostemon confertus* (R. Brown) Peter G. Wilson & J. T. Waterhouse	Fruit	2.6 cm	NIL
PH056	*Pavetta hongkongensis* Bremekamp	Fruit	1.1 cm	H. Y. Wong 008
PH057	*Pterospermum heterophyllum* Hance	Fruit	6 cm	K. H. Wong 004
PH058	*Eucalyptus exserta* F.	Fruit	2 cm	T. Y. Siu 571

	Mueller			
PH059	*Avicennia marina* (Forsskål) Vierhapper	Fruit	3.1 cm	D. T. W. Lau 252
PH060	*Mallotus peltatus* (Geiseler) Müller Argoviensis	Fruit	1.3 cm	H. L. Wang 005
PH061	*Trema tomentosa* (Roxburgh) H. Hara	Fruit	0.5 cm	H. L. Wang 006
PH062	*Memecylon ligustrifolium* Champion ex Bentham	Fruit	1.6 cm	H. L. Wang 007
PH063	*Polyspora axillaris* (Roxburgh ex Ker Gawler) Sweet	Fruit	3.4 cm	H. L. Wang 008
PH064	*Phyllanthus cochinchinensis* (Loureiro) Sprengel	Fruit	0.8 cm	H. L. Wang 009
PH065	*Melastoma sanguineum* Sims	Fruit	2.4 cm	H. L. Wang 010
PH066	*Helicteres angustifolia* Linnaeus	Fruit	1.9 cm	H. L. Wang 011
PH067	*Styrax suberifolius* Hooker &	Fruit	1.6 cm	H. L. Wang

	Arnott			012
PH068	*Camellia crapnelliana* Tutcher	Fruit	9.8 cm	Rare and endangered plants (J. Y. Y Lau, D. T. W. Lau, K. W. Lam, S. W. Shek) 027
PH069	*Camellia oleifera* C. Abel	Fruit	3.1 cm	Rare and endangered plants (D. T. W. Lau, J. Y. Y. Lau, S. W. Shek, K. W. Lam) 028
PH070	*Lithocarpus glaber* (Thunberg) Nakai	Fruit	7 cm	T. Y. Siu 687
PH071	*Cyclobalanopsis pachyloma*	Fruit	2.8 cm	T. Y. Siu 702

	(Seemen) Schottky			
PH072	*Callerya nitida* (Bentham) R. Geesink Leiden	Fruit	11.4 cm	H. L. Wang 014
PH073	*Wikstroemia indica* (Linnaeus) C. A. Meyer	Fruit	1.1 cm	H. L. Wang 015
PH074	*Reevesia thyrsoidea* Lindley	Fruit	3.4 cm	H. L. Wang 016
PH075	*Garcinia oblongifolia* Champion ex Bentham	Fruit	3.7 cm	H. L. Wang 017
PH076	*Strychnos angustiflora* Bentham	Fruit	2.6 cm	H. L. Wang 018
PH077	*Strychnos angustiflora* Bentham	Seed	1.2 cm	H. L. Wang 018
PH078	*Dianella ensifolia* (Linnaeus) Redouté	Fruit	1.1 cm	H. L. Wang 019
PH079	*Tetracera sarmentosa* (Linnaeus) Vahl	Fruit	1.2 cm	H. L. Wang 020
PH080	*Uvaria calamistrata* Hance	Fruit	6 cm	H. L. Wang 021

PH081	*Uvaria calamistrata* Hance	Seed	0.9 cm	H. L. Wang 021
PH082	*Hibiscus tiliaceus* Linnaeus	Fruit	3.3 cm	H. L. Wang 022
PH083	*Caesalpinia bonduc* (Linnaeus) Roxburgh	Fruit	8.4 cm	H. L. Wang 023
PH084	*Caesalpinia bonduc* (Linnaeus) Roxburgh	Seed	1.9 cm	H. L. Wang 023
PH085	*Vitex rotundifolia* Linnaeus f.	Fruit	0.7 cm	H. L. Wang 024
PH086	*Canavalia* sp.	Fruit	11 cm	H. L. Wang 025
PH087	*Canavalia* sp.	Seed	1.8 cm	H. L. Wang 025
PH088	*Ricinus communis* Linnaeus	Fruit	2 cm	H. L. Wang 026
PH089	*Ipomoea pes-caprae* (Linnaeus) R. Brown	Fruit	2.7 cm	H. L. Wang 027
PH090	*Psychotria asiatica* Linnaeus	Fruit	0.9 cm	H. L. Wang

				028
PH091	*Clerodendrum fortunatum* Linnaeus	Fruit	2.2 cm	H. L. Wang 029
PH092	*Brucea javanica* (Linnaeus) Merrill	Fruit	1.7 cm	H. L. Wang 030
PH093	*Antidesma bunius* (Linnaeus) Sprengel	Fruit	2.5 cm	H. L. Wang 031
PH094	*Gnetum luofuense* C. Y. Cheng	Seed	5.6 cm	M. C. Li 071
PH095	*Antidesma japonicum* Siebold & Zuccarini	Fruit	2.2 cm	M. C. Li 076
PH096	*Castanopsis eyrei* (Champion ex Bentham) Tutcher	Fruit	4.1 cm	M. C. Li 077
PH097	*Aidia canthioides* (Champion ex Bentham) Masamune	Fruit	1.5 cm	H. L. Wang 032
PH098	*Mallotus paniculatus* (Lamarck) Müller	Fruit	1 cm	H. L. Wang 033
PH099	*Strychnos umbellata*	Fruit	1.1 cm	H. L. Wang

	(Loureiro) Merrill			034
PH100	*Strychnos umbellata* (Loureiro) Merrill	Seed	0.9 cm	H. L. Wang 034

The carpological specimens no., species name, carpological parts, size and voucher specimens no. (if any) of the carpological material were documented.

Fig 4. A pipeline for 3D digitization of carpological material. (a) The set-up of the image capturing system includes background color, lighting, camera position and the position of the carpological material. The adjustment of these parameters depends on the morphology of the carpological material. (b) Image capturing process of each material usually lasts for 5 to 10 minutes. (c) The images are imported to the software for 3D model reconstruction. The set-up should be adjusted if the reconstruction was failed. (d) Post-reconstruction editing would be carried out if the model is successfully reconstructed.

The size of the carpological materials varied greatly, ranging from about 3 mm to 12 cm in length. They were diverse in fruit types, including nuts, aggregated follicles, schizocarps, drupes, capsules, legumes, berries etc. The well-established standard operation procedures and the 3D models significantly enabled the establishment of a

virtual carpological collection. With a user-friendly model viewer, a public database namely "Virtual Carpological Herbarium of Fruits and Seeds" (https://syhuherbarium.sls.cuhk.edu.hk/collections/3d-specimen/) was established for archiving virtual carpological collection. Since the database is a responsive webpage, the interfaces can function properly in various kinds of mobile devices or computers. The 3D models can be viewed with a HTML5 browser without any software installation in the user devices. All 3D models in the database can be freely browsed in 360° by simple mouse gestures on desktop computers or by fingers on mobile devices. In order to browse the virtual specimens smoothly, some hardware and software configurations are highly recommended: For desktop or laptop computers, CPU: Intel i5 2.1G or above; RAM: 6G or above; 3D GPU: CUDA enabled graphics card (e.g. Nvidia GeForce 1650 or above); Web browsers: Chrome 93.0.4577.82 or above / Firefox v92.0 or above / Edge v93.0.961.47 or above / Safari v5.1.7 or above were suggested. For mobile or tablet, OS: Android 9 or above/ iOS 14.8 or above were suggested.

Discussion

Advantages of the photogrammetric platform and the derived 3D models in this study

All the 3D models reconstructed by the photogrammetric platform were diverse in fruit types. Each has its unique morphological structure which can be observed clearly from all angles, such as the prickles on the fruit of *Xanthium* sp. (PH003) and the fruit of *Castanopsis lamontii* Hance (PH019 & PH045), the persistent involucre on the fruit of *Passiflora foetida* Linnaeus (PH034), and the grooves on the fruit of *Alpinia* sp. (PH050). These results demonstrated that the complex structures of carpological materials could be reconstructed by this photogrammetric platform. Most of these are important phenotypic traits for species identification and classification. Therefore, these 3D models could be used as important references in science and education.

The 3D models fully documented the carpological material at 360° view. Users can freely inspect the 3D models of the carpological material by simply dragging on the interface. Since a macro lens was used to capture the images, most of the macroscopic details could be clearly shown even when the 3D models were magnified up to 10 times. The structural fine details in 360° view would enable the users to inspect the 3D model that looks almost identical to the real carpological materials.

Instead of adding a scale bar during the post-reconstruction stage, a tailor-made scale bar was attached right below the carpological material during the image capturing stage as a reference of their realistic size, so all the 3D models include a carpological material and a scale bar which enabled an accurate size determination. Moreover, the photogrammetric platform is able to reconstruct carpological materials of wide range of size such as the seed of *Embelia* sp. (PH021) in 3 mm and the fruit of *Samanea saman* (Jacquin) Merrill J. Wash. (PH044) in 12 cm.

The color temperature of the light-plates was adjusted to 5600K which is identical to sunlight and the settings of the image capturing procedures were kept consistent. Hence, the final 3D models derived from the software reconstruction would not have color deviation, and truly reflected the genuine color of the specimens. Therefore, the 3D models of high color accuracy would become one of the prerequisites for species authentication.

Conventionally, it might be time-consuming and costly when scientists need to visit a distant herbarium for specimen inspection. Specimen loan could be helpful, but probably increase the risk of specimen damage. Currently, most virtual herbarium ha[illegible]

partially resolved the problems by providing 2D images of the specimens. However, the new photogrammetric platform in this study could revolutionize a new documentation of the carpological parts such as fruits and seeds which are crucial in authentication. Hence, the 3D model database could facilitate the usage of herbarium specimens across different regions and generations.

This platform has provided an efficient documentation of massive carpological collections. Only about 100 to 150 images were required to reconstruct a high-resolution 3D model, in which the reconstruction and editing of each 3D model required approximately an hour to complete. Hence, up to 10 3D models of carpological collections could be reconstructed per day according to the pipeline as shown in Fig 4, that is faster than other conventional methods as used in insect and animal documentation.

Resolved problems in this research

Light reflection from the material surface was the major problem during image capturing. The white spots reflected from light source could impair the image recognition by the software, and resulted in reconstruction failure. Nonetheless, this problem could be fixed by adding various kinds of diffusers such as white paper or soft

box to diffuse the light towards the carpological materials (Fig 5).

Fig 5. Light reflection on shiny surface. (a) Two light plates were placed on the left and right sides of the fruit of *Heritiera littoralis* Aiton (PH018) respectively. The light reflection created multiple white spots on the surface which resulted in failure of image recognition by the software. (b) A white paper was added between PH018 and the left light plate, which diffused most of the direct light towards it. The light reflection on the left side was significantly reduced.

On the other hand, the specimen orientation could significantly affect the result, and sometimes lead to reconstruction failure, especially in the long carpological material. The fruit of *Samanea saman* (Jacquin) Merrill J. Wash. (PH044) was used to illustrate the situation (Fig 6). Two sets of images were captured in horizontal and vertical orientation respectively. Results showed that only one side of PH044 was reconstructed when it was placed horizontally. The image depth of field could be a reason, because it was not feasible to get clear images from both ends of a long carpological material in horizontal position. Furthermore, the software might not be able to distinguish both ends of PH044 due to blurred image. A 3D model of long carpological material could be successfully reconstructed when it was placed in vertical

orientation, as the images are clearly enough to be recognized by the software (Fig 6).

Fig 6. Comparison of 3D models of long dimension carpological material reconstructed by different positioning. (a) A carpological material of the fruit of *Samanea saman* (Jacquin) Merrill J. Wash (PH044). (b)&(c) The 3D model when PH044 was placed horizontally. The structure on the front side was reconstructed but the back side was not, as shown by the regions with darker color. (d) Decrease in sharpness of the images of PH044 was found due to greater depth of field from two ends, which reduced the coherence between images so the software could not recognize its structure for further reconstruction. (e)&(f) The 3D model when PH044 was placed vertically. The structure was reconstructed completely on both sides.

Problems required further investigation

Although the results demonstrated that photogrammetry can be used to reconstruct 3D models of carpological materials, certain structures could not be reconstructed yet. Firstly, it was observed that the indumentum such as hairy or scaly coverings of fruits or seeds could not be reconstructed precisely. One possible reason was that the software misinterpreted the indumentum as flatten patterns on the surface instead of their tiny outgrowth structures. As a result, the indumentum could not be

reconstructed by the platform. Similar problems were found in reconstructing 3D models of birds or insect specimens by photogrammetry, in which the feathers or hairs were lost, or merged with the surface structures [16,19].

Secondly, long filament-like, very thin, and light structures could not to be reconstructed, because their small changes in physical positions due to rotation or air ventilation would result in discontinuity of the image recognition. Hence, the software treated the images as from different objects but not from the same one. Therefore, it cannot combine all of them for 3D model reconstruction.

Thirdly, semi-transparent structures could not be shown on the 3D models in this study. Instead, that parts would be stained with some background color, mainly because the software could not distinguish whether the parts are transparent or not, and simply adopted the background color that passing through the tissue. As a result, the reconstructed 3D model could not demonstrate the genuine texture and color of the material in different degree of transparency.

Comparison of the new platform to the existing 2D, 3D and CT scanning, and other photogrammetric platforms

2D specimen images

Both 2D scanning and the photogrammetric platform in this study document the specimens in digital images which would not exhibit physical or biological degradation as happened in the real specimens. However, images captured by 2D scanning only show a single side of the specimens which might not show some important characteristics. The photogrammetric platform in this study aims at documenting the reproductive structures of a plant, mainly the carpological parts, in 360° for authentication. Hence, the 3D models could provide additional information than those from the 2D images.

3D scanners

Most of the 3D scanners available on the market are bundled with default cameras, scanning platform and editing software, from which the equipment settings are fixed and not replaceable. They are designed for scanning objects within certain size range, usually ideal for objects ranging from 3 cm to 25 cm. Therefore, the compatibility and sustainability of these 3D scanners are limited. In contrast, the software and hardware in this photogrammetric platform are independent components and replaceable, so it allows further upgrade of any components. On the other hand, the settings adopted in this study are able to reconstruct carpological materials from 3 mm to 12 cm in

diameter, which have included a wide size range of fruits and seeds. In addition, this platform does not require calibration before image acquisition, but most of the 3D scanners do. Therefore, the photogrammetric platform and workflow in this study are more applicable and user-friendly.

CT scanning

CT scanning could reconstruct the specimen internal structures with numerous images, which can be stacked into 3D models, but they do not have colored surface and cannot reflect true phenotypic characteristics. Ijiri *et al*. [28] demonstrated a combination of CT scanning and image-based approach to create 3D models of insects and flowers with both internal structures and colored surface textures. However, the methodology required multiple steps and professional operations. In this study, the 3D models derived from the platform could also reflect the genuine color and phenotypic structures of the carpological materials, and the entire procedures are practically easier.

Other photogrammetric methods

Currently, a few 3D models of plant carpological parts by photogrammetric reconstruction were found on a public platform named Sketchfab

(https://sketchfab.com/disc3d/models), but massive database of digitized plant carpological materials was not yet found. On the other hand, more studies have been conducted in reconstructing 3D models of agricultural species to measure their size and growth rate. Therefore, the photogrammetric platform of this study was carpological materials specific, and it also provided 3D models with detailed morphology. Moreover, it was demonstrated to be user-friendly to reconstruct high quality 3D models from about 100 to 150 images.

3D sculpting

Compared to other mesh generation approaches of manual manipulation including sculpting and polygon drawing, the platform of this study serves the purposes of scientific documentation. Manual manipulation methods require sophisticated techniques in 3D modelling to create a lifelike model, but it does not sufficiently reflect the genuine features of the specimens. The artificial additions of characters would require detailed verification by professionals along the production. Hence, large-scale production of 3D models by sculpting techniques would be time-consuming and costly.

Application of 3D models in the research of carpological collection

Every specimen is a paramount scientific evidence for species identification and discovery, but damage and deterioration of the specimens could impair their authentication value, especially in type specimens. Specimens of over 100 years would probably have deteriorated morphology. Moreover, deformation and shrinking of some specimen parts are inevitable during the preparation procedures, and the specimens color also change over time. Hence, the specimen users could not differentiate which specimen characters are genuine or fake due to structure degradation. In contrast, 3D models reconstructed in this study could overcome these shortcomings and preserve the original form of the carpological materials in a digital version, which can be demonstrated by the following examples.

A local plant (*Sterculia lanceolata* Cavanilles) with follicle-type fruits was selected to explain this situation. Its specimen of over 50 years (Shiu Ying Hu 5569) and a 3D model of its fresh fruit (PH053) were compared (Fig 7). The general description of lived *S. lanceolata* fruits is red to bright scarlet in color, long ovate or ellipsoid, densely pubescent, base attenuate and apex beaked [29,30]. However, some of these characteristics were lost in the old specimen (Shiu Ying Hu 5569). The color of the specimen has already faded out. The fruit surface has a harder texture and numerous wrinkles. The changes of these features would give inaccurate evidences in

authentication. Alternatively, a 3D model of a freshly matured fruit of *S. lanceolata* (PH053) was reconstructed shortly after collection. The morphology of the fruit was well recorded in the 3D model that matched with the major characteristics including color and follicle shape as mentioned in Floras. Therefore, a good documentation of these 3D models in a database could be essential to botanists.

Fig 7. Comparison between 2D image and 3D models of fruits of *Sterculia lanceolata* Cavanilles. (a) A carpological material of the fruit of *S. lanceolata* (PH053). (b) 2D image of a specimen of *S. lanceolata* (Shiu Ying Hu 5569). (c) Front side of the 3D model of PH053. (d) Back side of the 3D model of PH053.

In addition, the 3D models reconstructed by this platform could help witness the morphological changes of the carpological structures at different time points, that is especially important in studying fruit maturation. Basically, the structural or caducous changes in various stages of drying fruits, such as detachment of stalk or calyx and even exocarp rupture would be recorded. Moreover, the seed dispersal process could also be documented in the 3D models. For instance, a series of 3D models of the fruit of *Lophostemon confertus* (R. Brown) Peter G. Wilson & J. T. Waterhouse (PH054A, PH054B, PH054C, PH054D) were reconstructed to record its morphological changes on

day 1, day 2, day 5 and day 10 respectively (Fig 8). On day 1, the fruit was not yet opened, enclosing all seeds. On day 2, the valves on the top of the fruit opened and started to disperse some seeds. On day 5, majority of the seeds had been released, and the 3-lobed capsule could be easily observed from the 3D model. On day 10, all the seeds had been released, and the wrinkles on the fruit surface become prominent. To conclude, the 3D models could provide significant evidences in continuous assessment of fruit development under specific time points.

Fig 8. 3D models of *Lophostemon confertus* (R. Brown) Peter G. Wilson & J. T. Waterhouse at different time points after collection. (a) A carpological material of the fruit of *L. confertus* was fixed for image capturing. (b) Top view of the 3D model (PH054) reconstructed on day 1. The valves on top of the fruit were closed. (c) Top view of the 3D model (PH054) reconstructed on day 2. The valves of the capsule started to retreat, and the seeds inside were exposed. Two sepals contracted and bended inwards. (d) Top view of the 3D model (PH054) reconstructed on day 5. Most of the seeds had fallen and the internal structure of the capsule was clearly seen. (e) Top view of the 3D model (PH054) reconstructed on day 10. All the seeds were released.

Future perspective from this research

The photogrammetric platform in this study is a prerequisite for the development of a virtual herbarium or museum with additional carpological information. A new database of more than 100 3D models of carpological collection was created. This study not only built up a good capacity of documenting a single seed or fruit, but also well recorded some infructescence, e.g. *C. lamontii* (PH045). This 3D model database could be incorporated as important archive of an herbarium. Hence, these plant specimens and carpological materials will no longer be only limited to professionals and researchers, but also be accessible and useful to the general public.

The deliverables from this project had contributed to the establishment of “Virtual Carpological Herbarium of Fruits and Seeds” in the Shiu-Ying Hu Herbarium (https://syhuherbarium.sls.cuhk.edu.hk/collections/3d-specimen/). All information of the database is presented with user-friendly interfaces in all desktop computers and mobile devices. It is expected that the database will facilitate basic science research and its application in plant authentication, species discovery and conservation.

References

1. Lughadha, EN, Miller C. Accelerating global access to plant diversity information. Trends Plant Sci. 2009;14(11): 622-628.
2. Beaman RS, Cellinese N. Mass digitization of scientific collections: new opportunities to transform the use of biological specimens and underwrite biodiversity science. ZooKeys. 2012;209: 7-17.
3. Blagoderov V, Kitching IJ, Livermore L, Simonsen TJ, Smith VS. No specimen left behind: industrial scale digitization of natural history collections. Zookeys;209: 133-146.
4. Barkworth ME, Murrell ZE. The US virtual herbarium: working with individual herbaria to build a national resource. ZooKeys. 2012;209: 55-73.
5. Tegelberg R, Mononen T, Saarenmaa H. High-performance digitization of natural history collections: automated imaging lines for herbarium and insect specimens. Taxon. 2014;63(6): 1307-1313.
6. Thiers BM, Tulig MC, Watson KA. Digitization of the new york botanical garden herbarium. Brittonia. 2016;68(3): 324-333.
7. Sweeney PW, Starly B, Morris PJ, Xu Y-M, Jones A, Radhakrishnan S, et al. Large-scale digitization of herbarium specimens: development and usage of an automated, high-throughput conveyor system. Taxon. 2018;67(1): 165-178.
8. Borges LM, Reis VC, Izbicki R. Schrödinger's phenotypes: Herbarium specimens show two-dimensional images are both good and (not so) bad sources of morphological data. Methods Ecol Evol. 2020;11(10): 1296-1308.
9. Klaus AV, Kulasekera VL. Schawaroch V. Three-dimensional visualization of insect morphology using confocal laser scanning microscopy. J Microsc. 2003;212(2): 107-121.
10. Klaus AV, Schawaroch V. Novel methodology utilizing confocal laser scanning microscopy for systematic analysis in arthropods (insecta). Integr Comp Biol. 2006;46(2): 207-214.
11. Kuzminsky, SC, Gardiner MS. Three-dimensional laser scanning: potential uses for museum conservation and scientific research. J Archaeol Sci. 2012;39(8): 2744-2751.
12. Stuppy WH, Maisano JA, Colbert MW, Rudall PJ, Rowe TB. Three-dimensional analysis of plant structure using high-resolution x-ray computed tomography. Trends Plant Sci. 2003;8(1): 2-6.
13. Brereton NJB, Ahmed F, Sykes D, Ray MJ, Shield I, Karp A, et al. X-ray micro-computed tomography in willow reveals tissue patterning of reaction wood and delay in programmed cell death. BMC Plant Biol. 2015;15(1): 1-12.

14. Nguyen CV, Lovell DR, Adcock M, La Salle J. Capturing natural-colour 3D models of insects for species discovery and diagnostics. PLoS One. 2014;9(4): e94346.
15. Ströbel B, Schmelzle S, Blüthgen N, Heethoff M. An automated device for the digitization and 3D modelling of insects, combining extended-depth-of-field and all-aide multi-view imaging. Zookeys. 2018;759: 1-27.
16. Brecko J, Mathys A. Handbook of best practice and standards for 2D+ and 3D imaging of natural history collections. Eur J Taxon. 2020;623: 1-115.
17. Postma M, Tordiffe ASW, Hofmeyr MS, Reisinger RR, Bester LC, Buss PE, et al. Terrestrial mammal three-dimensional photogrammetry multispecies mass estimation. Ecosphere. 2015;6(12): 1-16.
18. Giacomini G, Scaravelli D, Herrel A, Veneziano A, Russo D, Brown RP, et al. 3D photogrammetry of bat skulls: perspectives for macro-evolutionary analyses. Evol Biol. 2019;46(3): 249-259.
19. Medina JJ, Maley JM, Sannapareddy S, Medina NN, Gilman CM, McCormack JE. A rapid and cost-effective pipeline for digitization of museum specimens with 3D photogrammetry. PLoS One. 2020;15(8): e0236417.
20. Bot JA, Irschick DJ. Using 3D photogrammetry to create open-access models of live animals: 2D and 3D software solutions. In: Grayburn J, Lischer-Katz Z, Golubiewski-Davis K, Ikeshoji-Orlati V, editors. 3D/VR in the Academic Library: Emerging Practices and Trends [e-book]. Arlington: Council on Library and Information Resources; 2019 [cited 2021 Sep 15]: 54-72. Available from: https://www.clir.org/pubs/reports/pub176/
21. Aguilar MA, Pozo JL, Aguilar FJ, Sanchez-Hermosilla J, Páez FC, Negreiros J. 3D surface modelling of tomato plants using close-range photogrammetry. In: Chen J, Jiang J, Hans-Gerd MAAS, editors. XXIst ISPRS Congress Technical Commission V. 2008 Jul 3-11; Beijing, China. Germany: Copernicus Publications; 2008. p. 139-144.
22. Frasson RPdeM, Krajewski WF. Three-dimensional digital model of a maize plant. Agric For Meteorol. 2010;150: 478-488.
23. Ayob NZS, Kamarauzaman N, Sahriman N, Samad AM. Data acquisition for 3D surface modelling of chilli plant by using close range photogrammetry for volume estimation. In: 2015 IEEE Conference on Systems, Process and Control (ICSPC 2015). 2015 Dec 18-20; Bandar Sunway, Malaysia. Institute of Electrical and Electronics Enginners (IEEE); 2015. p. 162-167.
24. An N, Welch SM, Markelz RJC, Baker RL, Palmer CM, Ta J, et al. Quantifying time-series of leaf morphology using 2D and 3D photogrammetry methods for high-throughput plant phenotyping. Comput Electron Agric. 2017;135(1): 222-232.
25. Walter J, Edwards J, McDonald G, Kuchel H. Photogrammetry for the estimation

of wheat biomass and harvest index. Field Crops Res. 2018;216: 165-174.
26. Andújar D, Calle M, Fernández-Quintanilla C, Ribeiro Á. Dorado J. Three-dimensional modeling of weed plants using low-cost photogrammetry. Sensors (Basel). 2018;18(4): 1077.
27. Arief MAA, Nugroho AP, Putro AW, Dananta DH, Masithoh RE, Sutiarso L, et al. Three-dimensional (3D) reconstruction for non-destructive plant growth observation system using close-range photogrammetry method. IOP Conf Ser Earth Environ Sci. 2021;733(1): 012028.
28. Ijiri T, Todo H, Hirabayashi A, Kohiyama K, Dobashi Y. Digitization of natural objects with micro CT and photographs. PLoS One. 2018;13(4): e0195852.
29. Wu DL. *Sterculia lanceolata* Cav. In: Hong Kong Herbarium, South China Botanical Garden, editors. Flora of Hong Kong Volume 1. Hong Kong: Agriculture, Fisheries and Conservation Department (AFCD); 2007. p. 210.
30. eFloras. *Sterculia lanceolata* Cav [Internet]. St. Louis (MO): Missouri Botanical Garden, Cambridge (MA): Harvard University Herbaria; 2008 [cited 2021 Sep 6]. Available from: http://www.efloras.org/florataxon.aspx?flora_id=2&taxon_id=200013861

Click here to access/download;Figure;Fig1.tif

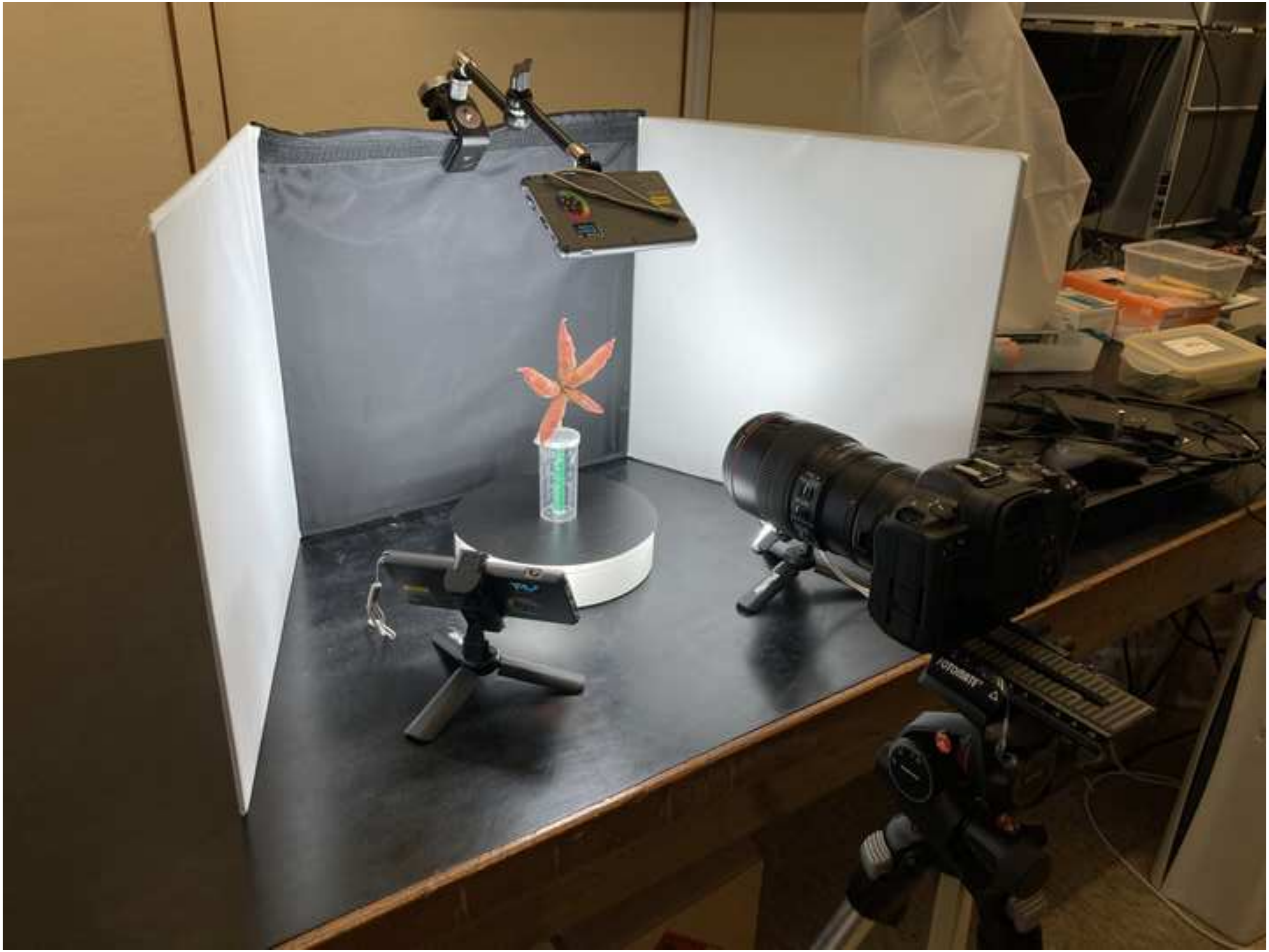

Click here to access/download;Figure;Fig2.tif

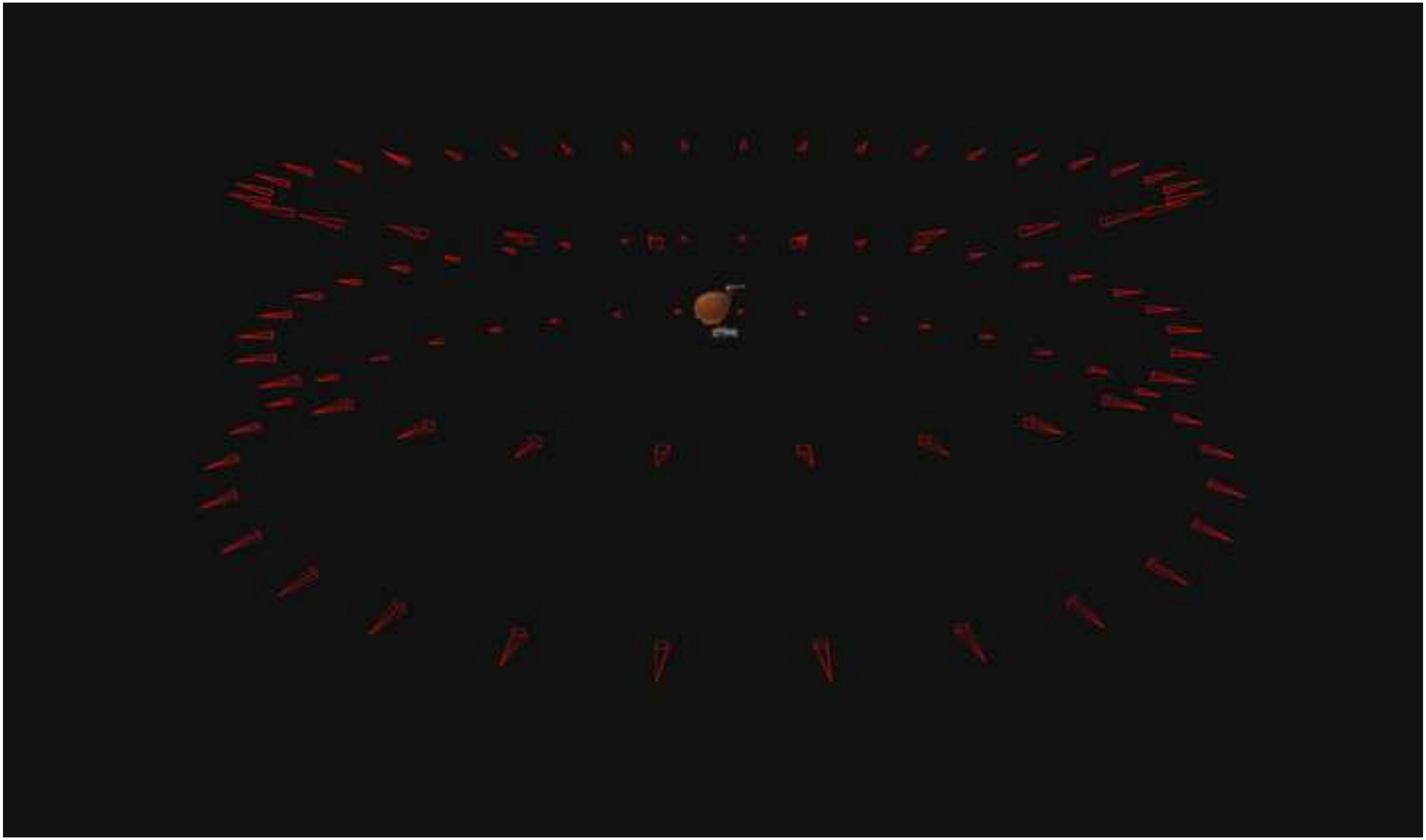

Click here to access/download;Figure;Fig3.tif

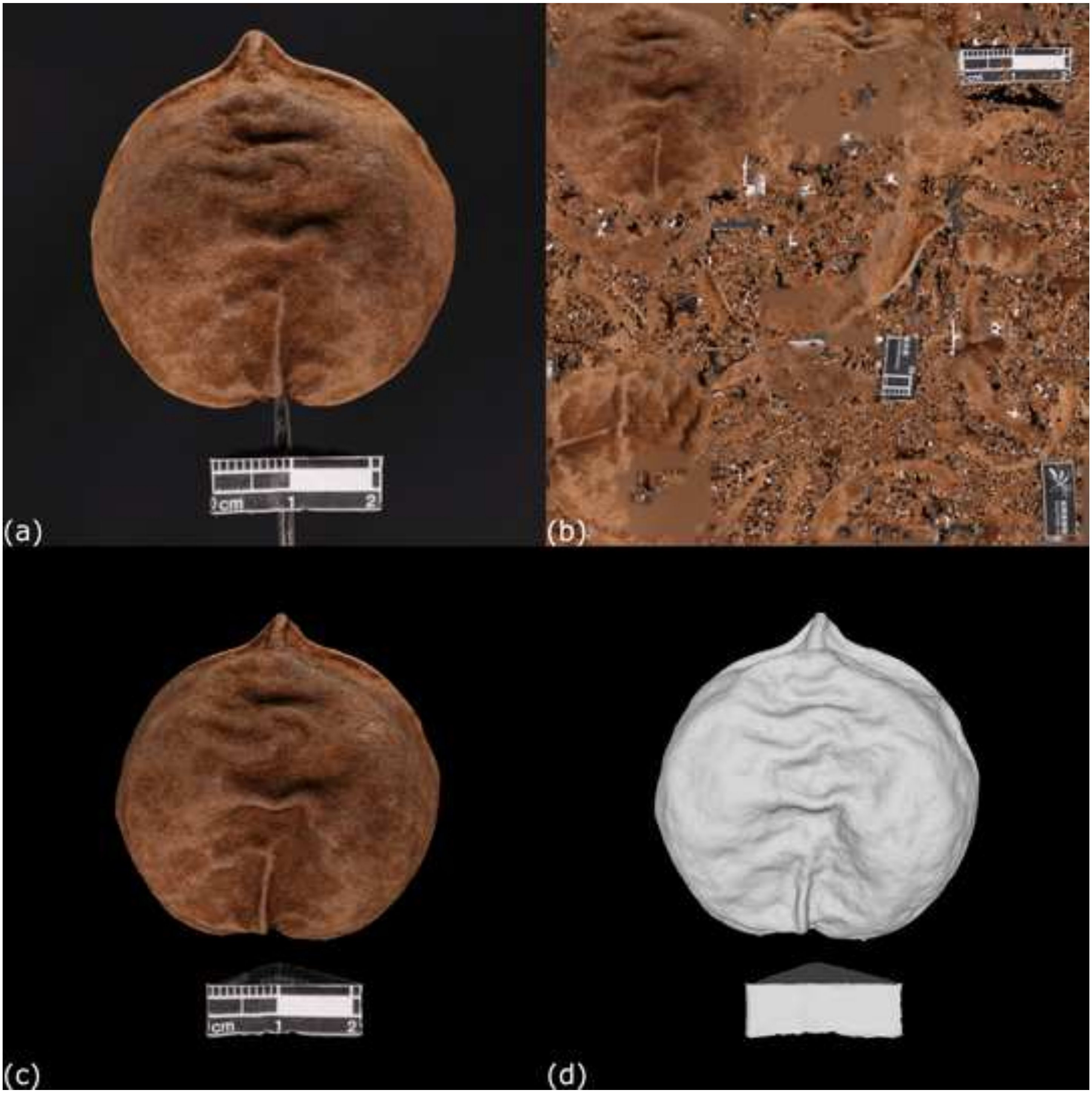

Click here to access/download;Figure;Fig4.tif

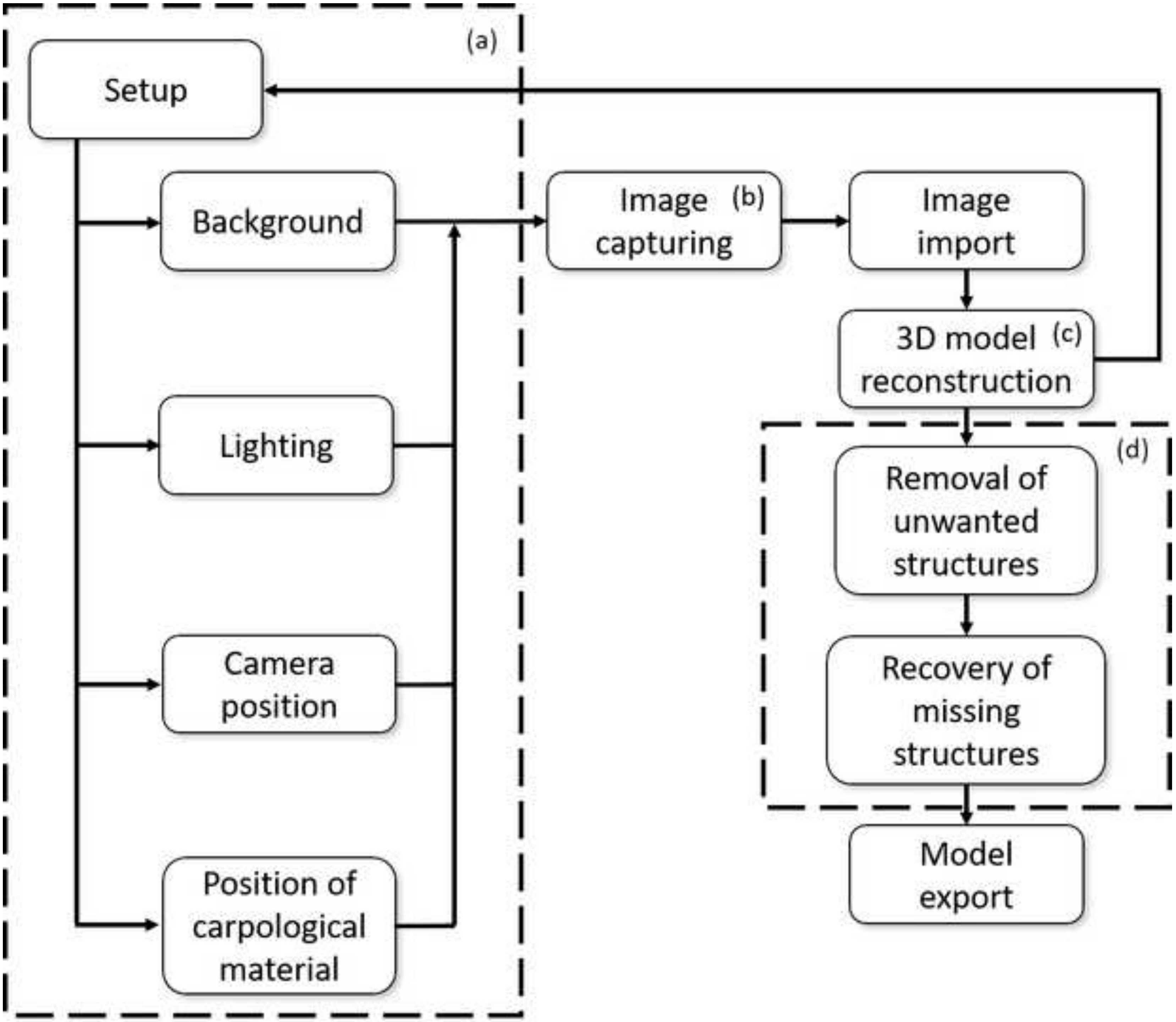

Click here to access/download;Figure;Fig5.tif

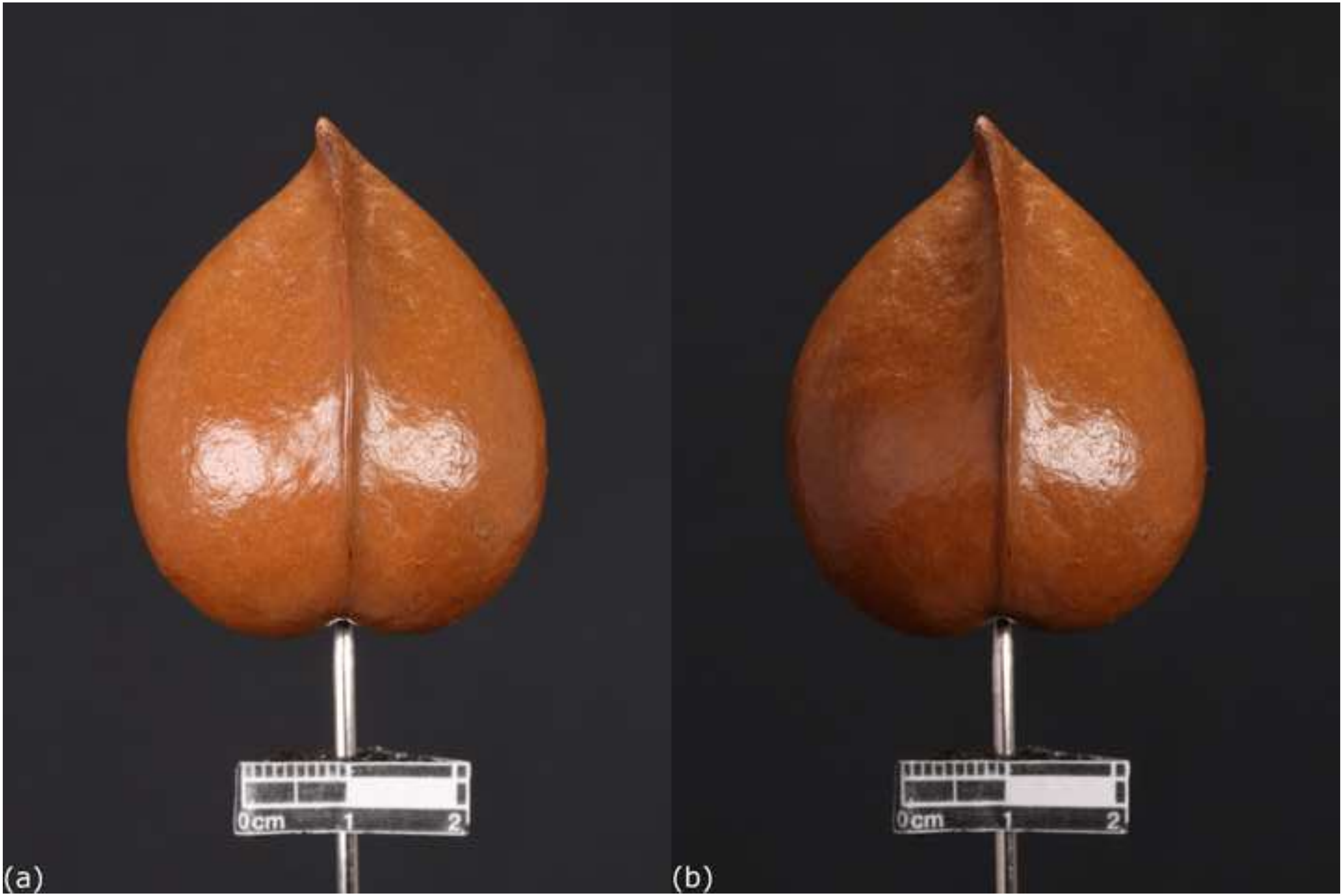

Click here to access/download;Figure;Fig6.tif

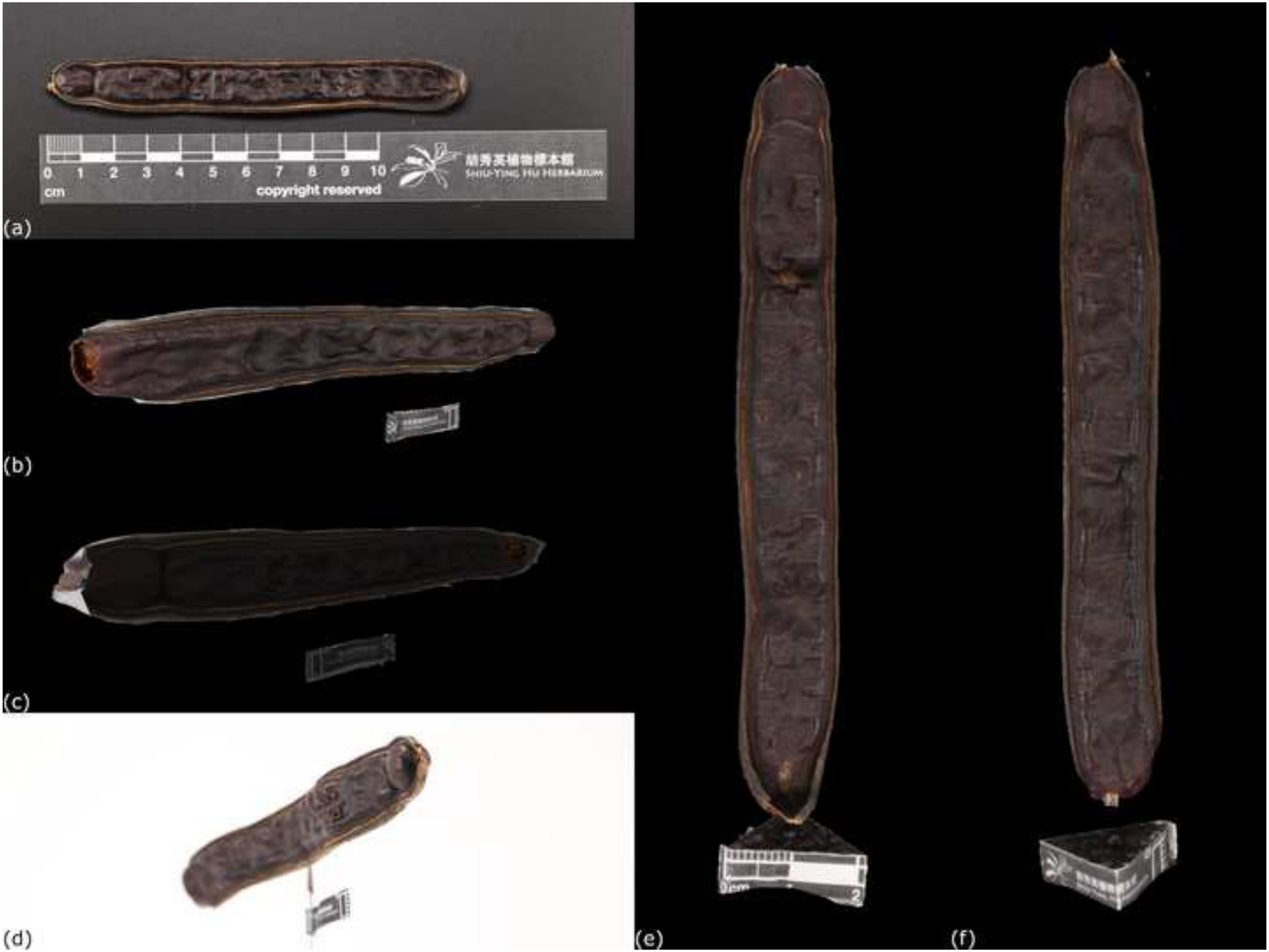

Click here to access/download;Figure;Fig7.tif

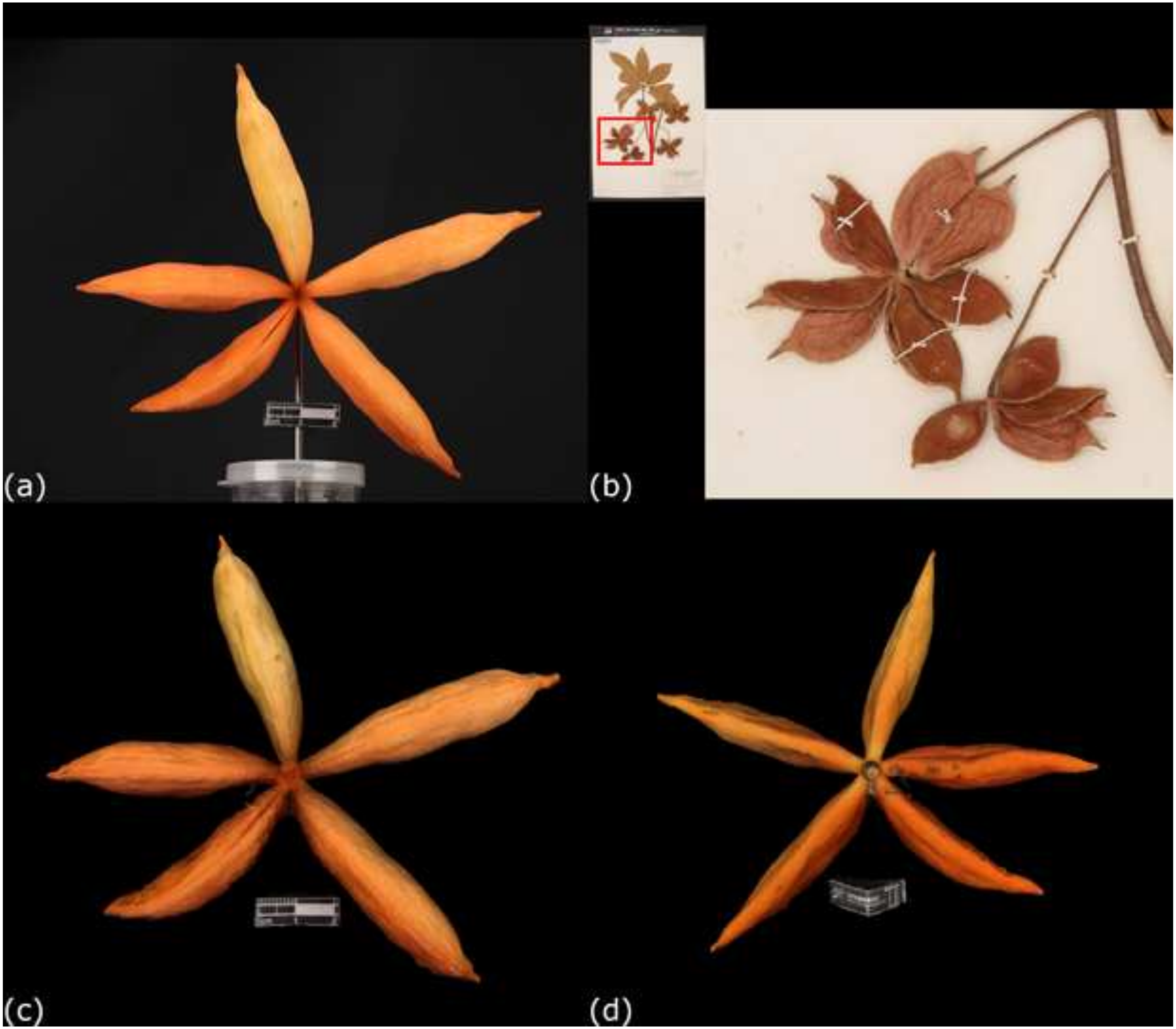

Click here to access/download;Figure;Fig8.tif

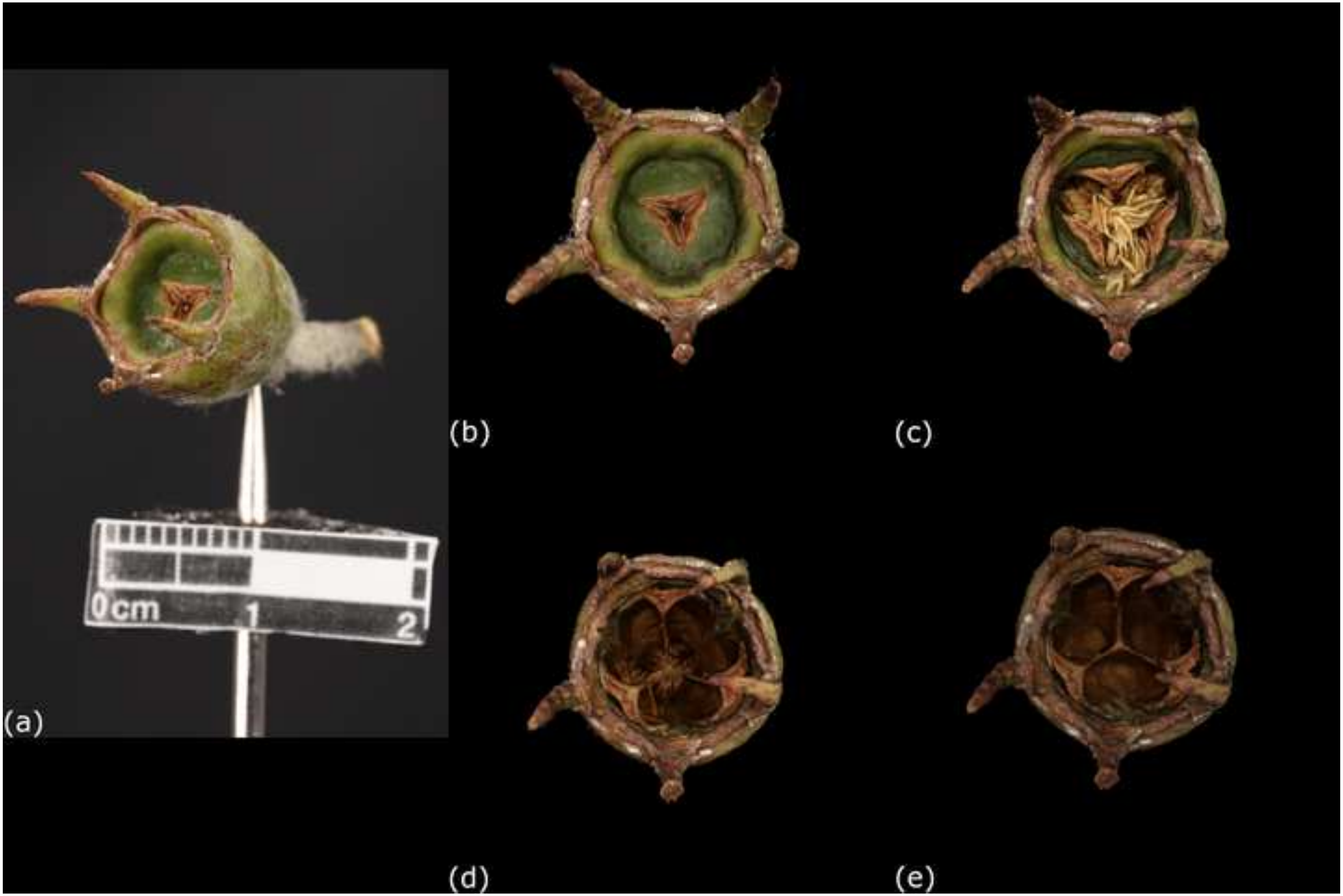

Supplement: Attachment — Submitted filename: Thiers comments_PONE-D-22-00891.pdf [file pone.0270199.s002.pdf]
