# Supplementary material for: Photogrammetric reconstruction of 3D carpological collection in high resolution for plants authentication and species discovery
Source: PLoS One. 2022 Aug 4;17(8):e0270199. doi: 10.1371/journal.pone.0270199 (PMC9352034; doi:10.1371/journal.pone.0270199)
Supplement: Attachment — Submitted filename: Response to Reviewers.docx [file pone.0270199.s003.docx]

Dear Editor,

**Rebuttal Letter**

I am sincerely writing to you to response all your comments in the Decision Letter in 24^th^ March 2022. Our replies are listed as below.

Comment 1. As suggested by Reviewer #1, for those who might be trying to decide whether or not to embark on the creation of such an image library, as part of a virtual herbarium, or some other project, it would be useful to have some additional information -- what is the image file size generated, what is the cost, or amount of time needed to capture a completed image.

**Reply:** Agreed. We have added some additional information and made some amendments in the section of discussion.

Comment 2. The arguments presented for why one should create such an imaging library could be strengthened – how are such images used in research today, both within the plant biodiversity community, and by outside users?

**Reply:** Agreed. Additional discussion has been added in the manuscript.

Comment 3. What are the key characteristics that can be observed through this method that cannot be seen with traditional 2D images?

**Reply:** 2D images usually show the front view, top view and side view of the carpological specimens. The key characteristic that can be observed in the 3D models was the 360° structure of the carpological materials which cannot be seen in 2D images. This is especially useful when inspecting carpological materials with complex structures.

Amendments were made in the section of discussion regarding to the comparison of 3D models to 2D images.

Comment 4. If the creation of a carpological 3D image library should indeed be a key component of a virtual herbarium, as the authors suggest, then there needs to be more of a discussion of how such images improve plant knowledge.

**Reply:** Agreed. Additional discussion has been added in the manuscript.

Comment 5. L36: “collection” should be “collections”.

**Reply:** Agreed and revised.

Comment 6. L71: “had also” should be “were also”.

**Reply:** Agreed and revised.

Comment 7. L76-85: Since this method was not successful, perhaps this section could be summarized more succinctly.

**Reply:** Agreed. We have summarized this section.

Comment 8. L79: “…were synchronized by a computer software under a same brand.” – Not sure what this means.

**Reply:** We agreed that this sentence does not explain clear enough. The computer software was published by the 3D scanner company which synchronize the 3D scanner and the digital camera for automatic photo taking. Yet, this section has been summarized and this sentence has been deleted.

Comment 9. L80: “could be placed” should be “was placed”.

**Reply:** Agreed. Yet, this section has been summarized and this sentence has been deleted.

Comment 10. L89: “had been” should be “were”.

**Reply:** Agreed and revised.

Comment 11. L91: “firstly used” should be “the first one used”.

**Reply:** Agreed and amended as follow.

“The digital camera Olympus TG-4 with a built-in microscope mode was the first one used in the experiment.”

Comment 12. L108: “had been” should be “were”.

**Reply:** Agreed and revised.

Comment 13. L122: “**Table 2**…” - I think this table is unnecessary.

**Reply:** We agreed that **Table 2** is unnecessary and removed it.

Comment 14. L128: “placed over” instead of “placed on top of”.

**Reply:** Agreed and amended as follow.

“Firstly, two lamps of no specification in light intensity and color temperature were placed over the carpological material.”

Comment 15. L150: “… the 8K-resolution images are too informative…” – Is this the best way to describe this?

**Reply:** We agreed that “informative” may not be the best way to describe the images. Amendment was made as follow.

“It was likely because the size of the 8K-resolution images is too large that is out of the software analytical range.”

Comment 16. L157-158: “…processing time was too long that might not be favorable to mass production.” – Suggestion: “…too long to be favorable for mass production.”

**Reply:** Agreed and amended as follow.

“…but the software processing time was too long to be favorable for mass production.”

Comment 17. L173: “It was possibly because…” – Suggest rewording: “Possibly this was because…”

**Reply:** Agreed and amended as follow.

“Possibly this was because PH003 was highly symmetric…

Comment 18. L300: “**Table 4**…” – Perhaps should be in supplemental material?

**Reply:** Agreed. **Table 4** has been moved to supporting information.

Comment 19. L304: “**Fig 4. A pipeline of** …” – I’d suggest calling this a workflow instead of a pipeline.

**Reply:** Agreed. “Pipeline” in L304 and elsewhere in the text were all amended.

Comment 20. L352: “…a tailor-made scale bar…” – Maybe use “customized” instead of “tailor-made”, which somehow just doesn’t sound right in this context.

**Reply:** Agreed and amended.

Comment 21. L369: “had” should be “have”.

**Reply:** Agreed and amended.

Comment 22. L373-374: Is this practical for a typical herbarium? What is the cost per seed imaged this way?

**Reply:** We believed that this method is practical for a typical herbarium. Additional information has been added, including the estimated cost of each 3D model.

Comment 23. L516: “lived” should be “living”.

**Reply:** Agreed and amended.

Comment 24. L560: “…is a prerequisite for the development…” – Perhaps to call this a “prerequisite” is a bit strong – certainly it would be beneficial.

**Reply:** We agreed that “prerequisite” is a bit strong and amended as follow.

“The photogrammetric platform in this study is beneficial to the development of a virtual herbarium or museum with additional carpological information.”

**Comments from Reviewer 1:**

This is a very interesting and potentially very useful contribution for the creation of 3D images of plant fruits and seeds. I believe that those interested in creating a collection of such images would find the information provided here very useful. For those who might be trying to decide whether or not to embark on the creation of such an image library, as part of a virtual herbarium, or some other project, it would be useful to have some additional information -- what is the image file size generated, what is the cost, or amount of time needed to capture a completed image. I think the arguments presented for why one should create such an imaging library could be strengthened -- how are such images used in research today, both within the plant biodiversity community, and by outside users? What are the key characteristics that can be observed through this method that cannot be seen with traditional 2D images? If the creation of a carpological 3D image library should indeed be a key component of a virtual herbarium, as the authors suggest, then there needs to be more of a discussion of how such images improve plant knowledge.

**Reply:** Thank you very much for your appreciation. We agreed with your suggestions and advice. Amendments has been done in the manuscript.

Comment 25. As suggested by Reviewer #2, the paper should focus on how to do plants authentication and species discovery using the reconstructed 3D model. Hopefully, the authors can add the above related content.

**Reply:** Agreed. Additional discussion has been added in the manuscript.

**Comments from Reviewer 2:**

This paper provides an accurate and efficient method to reconstruct detailed and high-resolution digital 3D models of carpological materials by photogrammetric method. It gives the method in details on selection on digital camera body, camera lens, adjustment of light source, and software selection. It is very like a guideline of 3D model reconstruction. And the readers can get the related information from other places, such as the guide of the 3D model software. But I think the paper should focus on how to do plants authentication and species discovery using the reconstructed 3D model. Hopefully, the authors can add the above related content.

**Reply:** Thank you very much for your appreciation. We agreed with your suggestions and advice. Further discussion has been added in the manuscript.

**Comments from Academic Editor:**

1. Please ensure that your manuscript meets PLOS ONE's style requirements, including those for file naming. The PLOS ONE style templates can be found at

<https://journals.plos.org/plosone/s/file?id=wjVg/PLOSOne_formatting_sample_main_body.pdf>  and

<https://journals.plos.org/plosone/s/file?id=ba62/PLOSOne_formatting_sample_title_authors_affiliations.pdf>

**Reply:** Agreed. We have updated our manuscript according to the style requirements.

2. In your Data Availability statement, you have not specified where the minimal data set underlying the results described in your manuscript can be found. PLOS defines a study's minimal data set as the underlying data used to reach the conclusions drawn in the manuscript and any additional data required to replicate the reported study findings in their entirety. All PLOS journals require that the minimal data set be made fully available. For more information about our data policy, please see <http://journals.plos.org/plosone/s/data-availability>.

Upon re-submitting your revised manuscript, please upload your study’s minimal underlying data set as either Supporting Information files or to a stable, public repository and include the relevant URLs, DOIs, or accession numbers within your revised cover letter. For a list of acceptable repositories, please see <http://journals.plos.org/plosone/s/data-availability#loc-recommended-repositories>. Any potentially identifying patient information must be fully anonymized.

Important: If there are ethical or legal restrictions to sharing your data publicly, please explain these restrictions in detail. Please see our guidelines for more information on what we consider unacceptable restrictions to publicly sharing data: <http://journals.plos.org/plosone/s/data-availability#loc-unacceptable-data-access-restrictions>. Note that it is not acceptable for the authors to be the sole named individuals responsible for ensuring data access.

We will update your Data Availability statement to reflect the information you provide in your cover letter.

**Reply:** Sorry. We didn’t mention the data set clearly in the manuscript. Amendment was made in the Results part and we have also added a table in the supporting information that shows the information of the 3D models.

3. We note that the grant information you provided in the ‘Funding information’ and ‘Financial Disclosure’ sections do not match. When you resubmit, please ensure that you provide the correct grant numbers for the awards you received for your study in the ‘Funding Information’ section.

**Reply:** The research work was supported by a donation fund from Wu Jieh Yee Charitable Foundation Limited. The fund has no formal grant number.

**Additional Editor Comments:**

Both reviews consider the paper to contain good points for publication. However, both of them still have several concerns to be addressed. Please address the comments and requests point by point. In addition, I think the authors should provide additional comparisons with competing techniques by including some quantitative evaluations, even in simple cases (or synthetic) could be enough. Regarding failure cases, I think they need to be deeply shown and discussed in the paper to help the reader. What about the use of photometric stereo approaches? I believe current methods could generate very accurate estimations in this context. Evaluation of accuracy from a theoretical point of view. Why should this method produce better solutions?

**Reply:** Thank you very much for your comments and suggestions. All the comments and requests from both reviewers have been addressed. We have also added figures and discussion related to the failure cases. We agreed that photometric stereo approaches produce more detailed topography than photogrammetry, but we have also considered its constraints when it comes to the reconstruction of carpological materials and its practicability in a typical herbarium. Further discussion has been added in the manuscript.

Thank you very much for your kindly reviews and consideration.

Yours sincerely,

Dr. David TW LAU

Curator of the Shiu-ying Hu Herbarium

School of Life Sciences

The Chinese University of Hong Kong
